# An efficient model for predicting human diseases through miRNA based on multiple-types of contrastive learning

**DOI:** 10.3389/fmicb.2023.1325001

**Published:** 2023-12-14

**Authors:** Qingquan Liao, Xiangzheng Fu, Linlin Zhuo, Hao Chen

**Affiliations:** ^1^College of Computer Science and Electronic Engineering, Hunan University, Changsha, China; ^2^School of Data Science and Artificial Intelligence, Wenzhou University of Technology, Wenzhou, China

**Keywords:** contrastive learning, diagnosis and treatment, graph collaborative filtering, human microbiota, miRNA-disease associations, sparse neighborhoods

## Abstract

Multiple studies have demonstrated that microRNA (miRNA) can be deeply involved in the regulatory mechanism of human microbiota, thereby inducing disease. Developing effective methods to infer potential associations between microRNAs (miRNAs) and diseases can aid early diagnosis and treatment. Recent methods utilize machine learning or deep learning to predict miRNA-disease associations (MDAs), achieving state-of-the-art performance. However, the problem of sparse neighborhoods of nodes due to lack of data has not been well solved. To this end, we propose a new model named MTCL-MDA, which integrates multiple-types of contrastive learning strategies into a graph collaborative filtering model to predict potential MDAs. The model adopts a contrastive learning strategy based on topology, which alleviates the damage to model performance caused by sparse neighborhoods. In addition, the model also adopts a semantic-based contrastive learning strategy, which not only reduces the impact of noise introduced by topology-based contrastive learning, but also enhances the semantic information of nodes. Experimental results show that our model outperforms existing models on all evaluation metrics. Case analysis shows that our model can more accurately identify potential MDA, which is of great significance for the screening and diagnosis of real-life diseases. Our data and code are publicly available at: https://github.com/Lqingquan/MTCL-MDA.

## 1 Introduction

MicroRNA (miRNA) is a kind of RNA molecule that is single-stranded and generally consists of 19–25 nucleotides (Cuperus et al., 2011), which is endogenous non-protein-coding, and highly conserved in evolution (Ambros, 2004). Relevant studies have shown that miRNA is involved in the human intestinal microbial environment, thereby affecting the pathogenesis of certain intestinal inflammation (Friedman et al., 2009). Some miRNAs bind to mRNA, thereby inhibiting activities such as mRNA degradation, causing the downregulation of mRNA expression function (Gebert and MacRae, 2019). Generally, miRNA exists in human peripheral blood, but it may also appear in intestinal fluid, saliva and other body fluids (Weber et al., 2010). Recent exploration has found that miRNA serves as a mediator to guide the interaction between cells and microbiota (Ji et al., 2018). In addition, certain metabolites produced by microorganisms can affect the expression of miRNA, thereby affecting the host's microecology. Some studies have verified that abnormal function of miRNA is involved in the pathogenesis of certain diseases (Park et al., 2017). Related studies have also revealed that miRNA and microbial communities are related to Inflammatory bowel disease (Qin et al., 2010; James et al., 2020).

MiRNA regulates the flow and expression of genetic information in space and time through post-transcriptional gene regulation or silencing (Zhang et al., 2018), which involves approximately 30 to 90 human genes (Cai et al., 2009). The ability of miRNAs to regulate apoptosis and growth of cells has been demonstrated by numerous studies (Neilson et al., 2007). Tumor formation often results when cells display abnormal growth and loss of apoptotic function (Hill and Tran, 2021). Although it is not completely clear how miRNAs regulate the development and maturation of nervous system and their physiological functions, it has been confirmed that the expression of miRNAs in nervous system is characterized by high time sequence, high conservation, and high specificity (Cao et al., 2016). At present, it is widely believed among researchers that miRNAs perform specific regulatory functions in the nervous system's development and operation. Many studies indicate that miRNAs are intricately involved in the precise regulation for the function and development of nervous system (Christensen and Schratt, 2009). Once the regulation is chaotic, it will inevitably lead to disease. Although the correlation between miRNAs and cerebrovascular diseases is still being explored, according to the analysis of relevant research data, miRNAs may also contribute to the development of cerebrovascular diseases (Hu et al., 2015). On one hand, miRNA can be used as a therapeutic target (Ganju et al., 2017) to achieve gene regulation (Weiland et al., 2012), and on the other hand, it can serve as a biomarker of disease diagnosis and disease screening (Mo et al., 2012).

The traditional methods for detecting miRNA mainly include Northern Analysis (Válóczi et al., 2004), Microarray (Li and Ruan, 2009), and Quantitative Real-time PCR (Benes and Castoldi, 2010). Northern Analysis (Válóczi et al., 2004) is a common method for detecting RNA based on hybridization (Várallyay et al., 2008), and it is one of the earliest methods for miRNA analysis. This method is simple and feasible, and most laboratories can operate without additional capital investment and equipment update. However, the analysis process requires a lot of manual operations, is not suitable for large-scale screening experiments, and it is usually unable to effectively distinguish miRNAs with small sequence differences. Microarray (Li and Ruan, 2009) also detects miRNA based on hybridization principle, which analyzes and understands the mechanism of miRNA expression regulation and gene expression regulated by miRNA by measuring the expression level of miRNA in a specific process. However, this method requires sufficient initial RNA samples, and it is difficult to clearly distinguish miRNAs with small differences, as well as precursor miRNAs with the same sequence and mature active miRNAs. Real-time quantitative PCR is a method that completes the overall analysis process by adding a fluorescent group to the DNA amplification reaction (Benes and Castoldi, 2010). However, it is of heavy workload and high cost. Through the implementation of reliable methodologies like these, researchers have established extensive bioinformatics databases that house experimentally verified miRNAs and their associations with diseases. These databases are widely acknowledged as dependable sources of information. For example, dbDEMC is a database of miRNAs related to human cancer (Xu et al., 2022). HMDD is a database of miRNA-related diseases (Huang et al., 2019). MiR2Disease is a miRNA-related disease database developed by Harbin University of Technology (Jiang et al., 2009). They provide a lot of valuable data support for future research.

The researches have proposed many computational methods to predict the MDAs well. Most of these approaches assume that miRNAs which have similar functions are potentially associated with diseases having similar phenotypes. For example, Chen and Yan (2014) applies regularization and semi-supervised strategies to predict miRNA-disease correlations. Jiang et al. (2010) proposes the network-based method to infer the potential MDAs. Luo systematically prioritizes disease-associated miRNAs using transduction learning-based collective prediction. Zhang et al. use a fast network link reasoning method based on linear neighborhood similarity. First, the known miRNA disease association is expressed as a binary network, and the miRNA is expressed as a correlation spectrum, so is the disease. Then the fast linear neighborhood similarity measure and the correlation curve are proposed to estimate the MDAs (Zeng et al., 2016). Huang integrates the nuclear similarity of miRNA-disease Gaussian interaction profiles into the original multiple data, and proposes a novel prediction model called PBMDA (You et al., 2017). By integrating multiple data, Chen et al. further propose an induction matrix strategy to forecast MDAs (Li and Ruan, 2009; Benes and Castoldi, 2010).

Recently, machine learning and deep learning have been leveraged in the field of biology, such as prediction of gene regulatory (Peng et al., 2022; Wang et al., 2022; Gao et al., 2023), drug discovery (Li et al., 2021a), and ncRNA protein interactions (Liu et al., 2022). Biomedical scientists are drawing inspiration from this approach, and utilizing machine learning algorithms to forecast possible correlations between miRNAs (microRNAs) and diseases, resulting in enhanced accuracy of prediction outcomes. Zhang et al. (2019) propose a new method which predictes MDAs using automatic encoding machines and extractes features based on unsupervised rule. Fu presentes a deep learning ensemble model, named DeepMDA, that leverages stacked autoencoders to extract complex features from similarity data. Ding et al. (2021) developes a deep learning model, known as VGAE-MDA, which is based on variational autoencoding of graphs and is intended for forecasting MDAs.

Due to the great progress of graph neural network (GNN)s on graph-structured data (Cai et al., 2021; Wang et al., 2022, 2023), GNN-based models have been developed to forecast miRNA-disease correlations. Wang and Chen (2023) estimate between miRNAs-diseases correlations using a hybrid model that combines graph convolutional networks and convolutional neural networks, which are boosted by multi-channel attention. Li et al. (2021b) suggest a model named GAEMDA, that utilizes autoencoder-based GNNs to recognize miRNA-disease correlations. Moreover, Li et al. (2022b) integrate attention mechanism into a hierarchical GNN to predict miRNA-disease correlations.

The above methods have proven their success, but they do not fully consider the sparseness of node neighborhoods, including node topological neighborhood sparseness and node semantic neighborhood sparseness that degrade the performance of these models. In this study, we propose a graph collaborative filtering based model that integrates a contrastive learning strategy for topological neighbors of miRNA (or disease) nodes and a contrastive learning strategy for semantic neighbors of nodes. Our model can well alleviate the node sparse neighborhood problem and accurately predict miRNA-disease correlations. Experimental results prove the superiority of our model to predict the association between miRNAs and diseases. Our contributions are summarized as:

1. We propose a novel method to predict miRNA-disease associations, integrating a contrastive learning strategy into a graph collaborative filtering.
2. We propose a contrastive learning strategy based on the topological neighbors of miRNA (or disease) nodes, which can be used to fully mine the topological information in the miRNA-disease bipartite graph.
3. The semantic similarity between miRNA and disease is used to enrich the neighborhood information of nodes in the miRNA-disease bipartite graph, and a contrastive learning strategy for node semantic neighbors is proposed. This reduces the noise impact brought by the contrastive learning strategy of node topological neighbors, while alleviating the problem of sparse neighborhoods of nodes in the dataset.
4. Based on the MDAs datasets, we have constructed multiple sets of comparative experiments to evaluate the effectiveness and stability of our model. And the corresponding case analysis proves that the MTCL-MDA model can provide a certain degree of advice for early intervention in diseases.

## 2 Materials and methods

Based on the graph collaborative filtering model, we apply contrastive learning strategies to node topological neighbors and node-based semantic neighbors, respectively, and propose a model named MTCL-MDA. In general, it is easy to aggregate topological neighbors in GNN-based research. However, for semantic-based neighbors, although various methods have been tried, the results are not satisfactory. And when the topological neighborhood of nodes is sparse, semantic information (such as feature vectors) becomes more critical for the accurate representation of nodes. Therefore, the importance of semantic neighbors is self-evident. In our study, contrastive learning strategies are applied in two aspects to alleviate this problem, and the final experimental results are also very satisfactory. We present our approach in the following sections.

### 2.1 Problem formulation

Our main goal is to predict unknown MDAs based on observed MDAs. These known MDAs can be used as the basis for constructing a bipartite graph G = (M, D, A). M represents miRNAs collection, D represents disease collection, and A represents MDAs.

Our main goal is to predict unknown MDAs based on observed MDAs. These known MDAs can be used as the basis for constructing a bipartite graph *G* = (*M, D, E*). *M* = {*m*_1_, …, *m*_*N*_} represents miRNAs collection, *D* = {*d*_1_, …, *d*_*N*_} represents disease collection, and *A* represents MDAs. If there is a link from node *m*_*i*_ to *d*_*j*_, *A*_*ij*_ = 1, otherwise *A*_*ij*_ = 0.

Predicting MDAs involves node neighborhood information. $N^{h}\left(v_{i}\right)$ represents the set of neighbors of node *v*_*i*_ within *h*-order. In our research, we focus on the unweighted graph, *v*_*i*_ is regarded as the target node and $v_{j}\in N^{h}\left(v_{i}\right)$ is the neighbor within the *h*-order.

Link prediction problems (Lü and Zhou, 2011) are divided into time link prediction (predicting potential new links in evolutionary networks) and topological link prediction (inferring unknown links in static networks). Similar to the latter, given partial observations of the topology, it is expected to predict unknown links. In practical problems, given the correlation topological structure of some observed miRNAs and diseases, we can predict the unknown correlation, that is, whether the miRNA is related to the disease.

### 2.2 Model architecture

**Figure 1:**
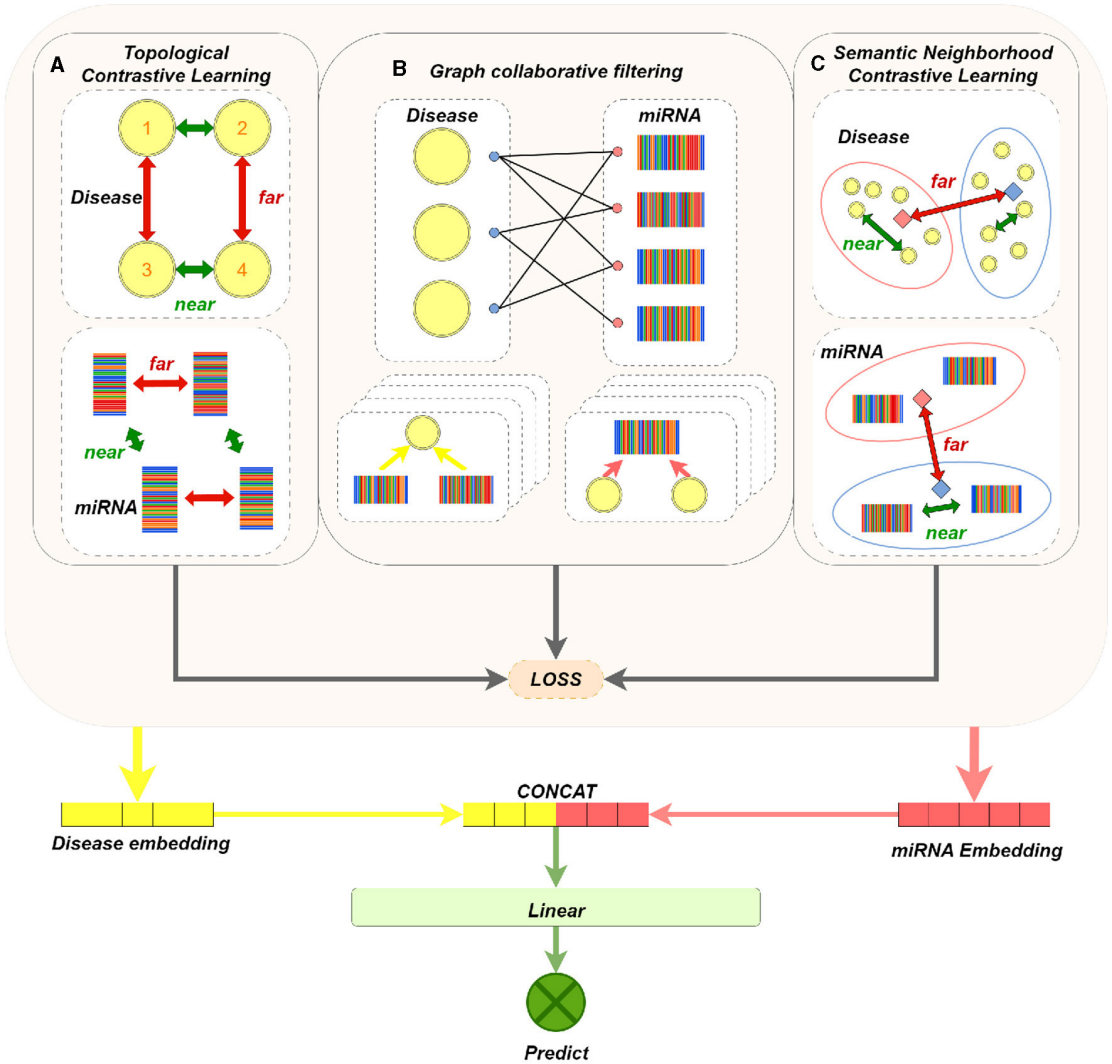
MTCL-MDA model architecture figure. **(A)** The dashed box on the left shows the contrastive learning of disease nodes based on topological neighborhood. For a disease node 1, its embedding and the embedding obtained in the even layer of GNN constitute a positive sample; its embedding in the even layer constitutes a negative pair with the embedding of other disease nodes not connected to it in the even layer. Conduct contrastive learning training so that the distance between nodes with similar structure (such as 1 and 2, 3 and 4) is smaller, and the distance between 1 (2) and 3 (4) with dissimilar structure is larger. **(B)** Brief process of representative graph collaborative filtering, where four layers of aggregation operations are performed. **(C)** The dashed box on the left represents the contrastive learning of disease nodes based on semantic neighborhood. The orange and blue solid squares represent the cluster centers, the nodes in the cluster and the cluster centers constitute positive samples, and the nodes in the cluster and other cluster centers constitute negative samples. In this way, contrastive learning training is carried out, so that the distance between nodes with similar feature vectors is smaller, and the distance between nodes with dissimilar feature vectors is larger. Processing on miRNA nodes is similar to disease nodes.

Our model mainly consists of three parts: graph collaborative filtering, contrastive learning of miRNA (or disease) nodes, and MDAs prediction. The main components of the model are shown in Figure 1. We apply graph collaborative filtering to disseminate network information in the miRNA-disease bipartite graph, and update node embeddings by aggregating neighbors. And the model integrates the contrastive learning strategy of topological neighbors and the contrastive learning strategy of semantic neighbors. Immediately, it can not only reduce the negative impact of noise caused by purely using topology contrastive learning strategy, but also alleviate the problem of sparse topological neighborhood of nodes. Finally, we concatenate the output miRNA and disease node embeddings to obtain a paired vector, and then feed it into MLPs to determine whether there exists a association between miRNA and disease.

### 2.3 Graph collaborative filtering

As mentioned at the beginning of this section, GNN-based approaches generate miRNA (or disease) embeddings by applying propagation and prediction functions to the topology composed of MDAs. In this study, a graph collaborative filtering model is employed to complete the propagation process. Specifically, Our propagation function is as follows:


(1) $$\begin{array}{l} X_{m}^{l+1}=\underset{d\in N_{m}}{\sum }\frac{1}{\sqrt{\left|N_{m}||N_{d}\right|}}X_{d}^{l} \end{array}$$

(2) $$\begin{array}{l} X_{d}^{l+1}=\underset{d\in N_{d}}{\sum }\frac{1}{\sqrt{\left|N_{d}||N_{m}\right|}}X_{m}^{l}, \end{array}$$

where $X_{m}^{l}$ and $X_{d}^{l}$ denote the embeddings of miRNA and disease nodes on *l*_*th*_-layer network, respectively. *N*_*m*_ and *N*_*d*_ denote the neighbor sets of nodes *m* and *d*, respectively. After the *L* layer propagation, We utilize the weighted *sum* function as the readout function and aggregate the representations from all layers to derive the ultimate representation, as depicted below:


(3) $$\begin{array}{l} X_{m}=\frac{1}{L+1}\overset{L}{\underset{l=0}{\sum }}X_{m}^{k}, \end{array}$$

(4) $$\begin{array}{l} X_{d}=\frac{1}{L+1}\overset{L}{\underset{l=0}{\sum }}X_{d}^{k}, \end{array}$$

where *X*_*m*_ and *X*_*d*_ respectively represent the final node embedding of nodes *m* and *d*. And then calculate the inner product between nodes *m* and *d* as the correlation score:


(5) $${\hat{y}}_{m,d}=X_{m}{\;}^{T}X_{d},$$

where ${\hat{y}}_{m,d}$ represents the predicted score of (*m, d*) pair. The BPR loss function (Rendle et al., 2012) is employed to train direct MDAs. Specifically, the loss function can make positive MDAs scores tend to be larger than negative association scores. Formally, the objective function of BPR loss is as follows:


(6) $$\begin{array}{l} L_{BPR}=\underset{\left(m,i,j\right)\in O}{\sum }-\log\sigma \left({\hat{y}}_{m,i}-{\hat{y}}_{m,j}\right), \end{array}$$

where *L*_*BPR*_ and σ represent *BPR* loss and *sigmod* activation, respectively. *O* = {(*m, i, j*)|*R*_*m, i*_ = 1, *R*_*m, j*_ = 0} represents the paired training data, and *j* indicates the sampled diseases with which miRNA *m* is not associated. By optimizing *L*_*BPR*_, our proposed model can model these observed MDAs. However, the higher-order neighbors within miRNA (or disease) is also valuable for prediction task. For example, miRNAs within the same cluster have a high probability of being linked to the same miRNAs (or diseases). Next, we demonstrate two contrastive learning strategies to capture the respective latent neighbor relationships of miRNAs and disease nodes.

### 2.4 Contrastive learning strategy based on topological neighborhood

This subsection will demonstrate a contrastive learning strategy based on miRNA (or disease) nodes. More specifically, each miRNA (or disease) node is learned contrastively with its own topological neighbors. Easily, the node embeddings of these neighbors can be aggregated by GNN propagation. Bipartite graphs are formed on direct MDAs, and even layer propagation naturally gathers information from homogeneously structured neighbors. For example, node representations of similar neighbors can be obtained from the output of even-numbered layers (such as 2, 4, 6) based on GNN models. We regard the embedding of the miRNA node itself and its corresponding output embedding in even layers as positive samples. Based on the InfoNCE (Aitchison, 2021) loss function, we propose a structure-contrastive learning objective as follows:


(7) $${ℒ}_{S}^{M}=\sum_{m\in M}-\log\frac{\exp\left(\left(X_{m}^{\left(k\right)}\cdot X_{u}^{\left(0\right)}/\tau \right)\right)}{\sum_{d\in M}\exp\left(\left(X_{m}^{\left(k\right)}\cdot X_{d}^{\left(0\right)}/\tau \right)\right)}.$$

*M* means the set of miRNA nodes. $X_{m}^{\left(k\right)}$ is the normalized output of *k*_*th*_ layer in GNN, *k* is an even number, and τ is the temperature hyperparameter on the numerator and denominator in the softmax function. Similarly, the topological neighbor contrastive learning strategy for disease nodes can be summarized as follows:


(8) $${ℒ}_{S}^{D}=\sum_{d\in D}-\log\frac{\exp\left(\left(X_{d}^{\left(k\right)}\cdot X_{u}^{\left(0\right)}/\tau \right)\right)}{\sum_{m\in D}\exp\left(\left(X_{d}^{\left(k\right)}\cdot X_{d}^{\left(0\right)}/\tau \right)\right)}.$$

where *D* means the set of disease nodes.

Integrating the topological neighbor contrastive learning strategy of miRNA nodes and the topological neighbor comparison learning strategy of disease nodes, the following can be obtained:


(9) $$\begin{array}{l} L_{S}=L_{S}^{M}+\alpha L_{S}^{D}. \end{array}$$

where α represents the adjustment parameter for two losses.

### 2.5 Contrastive learning strategy based on semantic neighborhood

As mentioned in Section 2.4, the topological contrastive learning strategy focuses on the neighbors defined by the miRNA-disease bipartite graph. However, it only takes into account the loss of contrast between a miRNA (or disease) node and its homogeneous neighbors. Meanwhile, it indiscriminately computes the contrastive loss of miRNA (or disease) nodes, which will inevitably introduce noise information. To mitigate suffering from topological neighbor noise, we consider extending the contrastive learning strategy by incorporating miRNA (or disease) node semantic neighbors. For a miRNA (or disease) node, its semantic neighbors refer to nodes that are unreachable on the miRNA-disease bipartite graph but have similar feature vectors.

Motivated by previous work (Mirman, 2011), we can determine semantic neighbors by learning latent prototypes of nodes. Therefore, we construct a prototype-based contrastive learning objective to identify potential semantic neighbors of miRNA (or disease) nodes. Meanwhile, the semantic neighbor-based contrastive learning strategy is integrated into the whole contrastive learning framework to better capture the semantic features of miRNAs (or diseases). Specifically, miRNA (or disease) nodes with similar node embeddings will be assigned into the same clusters using a clustering algorithm. These clusters are represented by the central nodes of the clusters, which are called prototypes. The process can use the EM learning algorithm (Kushary, 1998) to optimize the proposed prototype-contrastive learning function. Optimized by maximizing the log-likelihood of the following probability distribution function:


(10) $$\sum_{m\in ℳ}\text{log }p\left(e_{m}|\Theta ,R\right)=\sum_{m\in ℳ}\log\sum_{c_{i}\in C}p\left(e_{m},c_{i}|\Theta ,R\right)$$

And Θ indicates all parameters, **R** indicates the miRNA-disease bipartite graph, and *c*_*i*_ is the potential comparison prototype of the miRNA node *m*. Similarly, we can define an optimization objective function for the set of diseased nodes:


(11) $$\sum_{d\in D}\text{log }p\left(e_{d}|\Theta ,R\right)=\sum_{d\in D}\log\sum_{c_{i}\in C}p\left(e_{d},c_{i}|\Theta ,R\right)$$

Applying the InfoNCE (Aitchison, 2021) loss function, we can optimize the following objectives based on contrastive strategies:


(12) $${ℒ}_{P}^{D}=\sum_{m\in M}-\log\frac{\exp\left(e_{m}\cdot c_{i}/\tau \right)}{\sum_{c_{t}\in C}\exp\left(e_{m}\cdot c_{t}/\tau \right)},$$

where *c*_*i*_ is the prototype node of miRNA node *m*, which is obtained by clustering all miRNA node embeddings by k-means algorithm. The set of miRNA nodes can be assigned into *k* clusters, and the value of *k* can be set as required. A similar process also applies to disease node sets:


(13) $${ℒ}_{P}^{D}=\sum_{d\in D}-\log\frac{\exp\left(e_{d}\cdot c_{j}/\tau \right)}{\sum_{c_{t}\in C}\exp\left(e_{d}\cdot c_{t}/\tau \right)},$$

where *c*_*j*_ represents the prototype node of the disease node *d*. By integrating the contrastive learning process of miRNA node sets and the contrastive learning process of miRNA node sets, we can get the final semantic neighbor-based optimization objective:


(14) $$\begin{array}{l} L_{P}=L_{P}^{M}+\alpha L_{P}^{D} \end{array}$$

According to this semantic neighbor-based contrastive learning strategy, on the one hand, it can reduce the impact of noise brought about by topology contrastive learning, and on the other hand, it can alleviate the problem of sparse node neighborhoods.

Therefore, the overall loss of the model is:


(15) $$\begin{array}{l} L=L_{BPR}+\beta_{1}L_{S}+\beta_{2}L_{P}+\beta_{3}||\Theta |{|}_{2} \end{array}$$

where β_1_, β_2_ and β_3_ are the parameter to control the weight, and Θ denotes all parameters of GNN model. We can apply EM algorithm to optimize the solution.

### 2.6 Prediction of MDAs based on MLP

We integrated the obtained embeddings of miRNA and disease nodes to further predict whether they are positive pairs or negative pairs. Commonly used integration methods include Hadamard product, vector inner product, vector addition, and concatenate operations. In this study, we empirically selected the concatenate operation:


(16) $${\hat{y}}_{m,d}=concatenate(X_{m},X_{d}),$$

where *X*_*m*_ and *X*_*d*_ represent the embeddings of miRNA *m* and disease *d*, respectively. If the miRNA *m* is associated with the disease *d*, then the (*m, d*) pair is positive, otherwise it is negative. The concatenated embedding representation *x* will be fed into MLPs, and finally output:


(17) $$\begin{array}{l} S\left(x\right)=\frac{1}{1+\exp\left(-x\right)}. \end{array}$$

Then the BCE loss (Wu et al., 2020) for the classification is calculated by:


(18) $$\begin{array}{l} loss=-y\log\left(S\left({\hat{y}}_{m,d}\right)\right)-\left(1-y\right)\log\left(1-S\left({\hat{y}}_{m,d}\right)\right), \end{array}$$

where *y* represents the true MDA in the dataset, and its value is 0 or 1. $S\left({\hat{y}}_{m,d}\right)$ represents the label predicted by the classifier.

### 2.7 Preliminary disease screening

**Figure 2:**
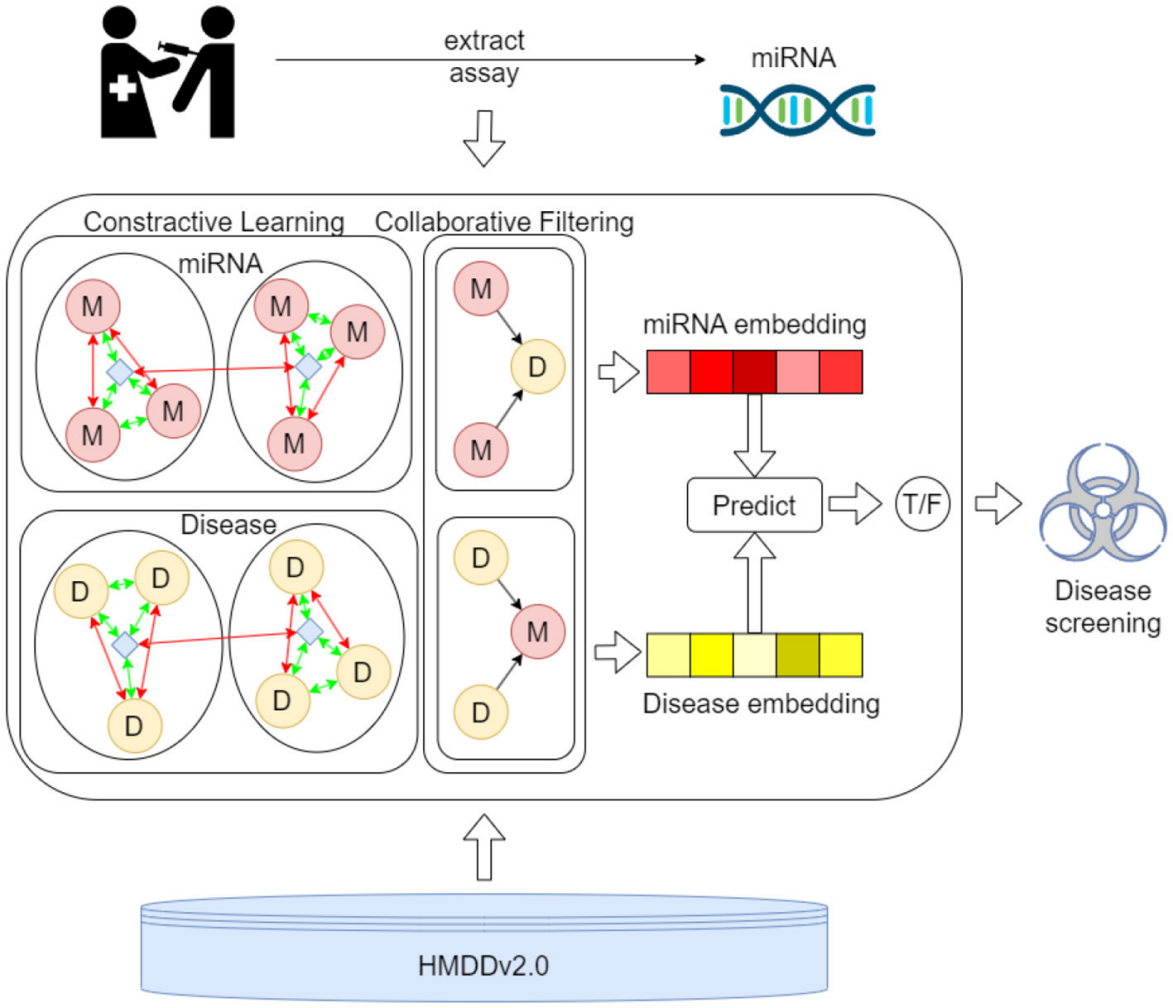
The process of the proposed MTCL-MDA model for preliminary disease screening.

Figure 2 presents the process of the proposed MTCL-MDA model for preliminary disease screening. First, samples are taken from the patient's relevant organs or tissues and assayed to extract key miRNA components. Then, the proposed model was used to predict diseases associated with this miRNA in the HMDD v2.0 database. The first process involves more complex biochemical testing and analysis. The proposed model can serve as a preliminary screening tool for the disease and play an active role in the second process. And our webserver is publicly accessible at: https://huggingface.co/spaces/ZZCrazy00/MDA.

## 3 Results

We use pytorch tool for building deep learning frameworks, to implement the MTCL-MDA model. To assess the effectiveness of the proposed model, we conducted extensive comparative experiments using the miRNA-disease dataset. This section mainly includes the following parts.

### 3.1 Datasets and experimental settings

We use datasets downloaded from HMDD v2.0 database. It contains 495 miRNAs, 383 diseases and 5,430 MDAs verified by experiments. The association information is represented by a matrix *A* with the size of (495, 383), where *A*_*ij*_ = 1 indicates that the *i*_*th*_ miRNA associated with the *j*_*th*_ diseaseand *A*_*ij*_ = 0 indicates that there is no association.

We evaluate the performance of the proposed model in terms of *AUC*, *Accuracy* (abbreviated as *ACC*), *Specificity* (abbreviated as *SPE*), *Precision* (abbreviated as *PRE*), *Recall* (abbreviated as *REC*), *F*1−*score*, and other metrics. The metrics used in our experiments are expressed as follows:


(19) $$\begin{array}{l} Acc=\frac{TP+TN}{TP+TN+FP+FN},\;Spe=\frac{TN}{TN+FP}, \end{array}$$

(20) $$\begin{array}{l} Sen=\frac{TP}{TP+FN},\;Pre=\frac{TP}{TP+FP},F1-score=2*\frac{Pre*Sen}{Pre+Sen} \end{array}$$

In the above equations, *TP* represents the positive association ratio of miRNA-disease correctly classified, *FP* represents the positive association ratio of miRNA-disease misclassified, *TN* represents the negative association ratio of miRNA-disease correctly classified, and *FN* represents misclassified miRNA-disease negative association ratio. And we compare the proposed model against the following eight baselines. WBSMDA (Chen et al., 2016) and RFMDA (Chen et al., 2018) integrates multiple similarity relations into a unified network to identify potential MDAs. PBMDA (You et al., 2017) integrates three interrelated networks and then predicted potential MDAs based on a depth-first search strategy. LLCMDA (Qu et al., 2018) is a method that utilizes locally constrained linear coding for predicting MDAs. EDTDA (Chen et al., 2019) is an innovative method that utilizes decision tree-based algorithms to predict MDAs. GBDT-LR (Zhou et al., 2020) combines gradient boosted decision trees and logistic regression to predict MDAs. MCLPMDA (Yu et al., 2019) predicts MDAs based on matrix completion. GAEMDA (Peng et al., 2022) predicts MDAs based on GNN and autoencoder.

### 3.2 Performance evaluation

We evaluate the performance of each model in terms of the metric *AUC* and the experimental results are reported in Table 1. Overall, all models achieved good results except the WBSMDA (Chen et al., 2016) model, which may be because most models have integrated and processed MDAs information. For example, the EDTMDA (Chen et al., 2019) and GBDT-LR (Zhou et al., 2020) models use an ensemble learning strategy, and the MCLPMDA (Yu et al., 2019) model uses a matrix completion strategy. We find that the GAEMDA (Li et al., 2021b) model achieves the second best results. The reason lies in it not only considers the topological information in the miRNA-disease bipartite graph in spite of fully considering the feature information of the nodes by using an autoencoder. It can also be seen from Table 1 that among all the models, the proposed MTCL-MDA performs best on *AUC* metric, and the reason is that the proposed MTCL-MDA considers not only the node topological information but also the topological neighborhood of nodes is sparse.

**Table 1:** Comparison of MTCL-MDA model with other advanced methods on AUC indicator.

**Models**	**AUC**
WBSMDA (Chen et al., 2016)	81.85%
RFMDA (Chen et al., 2018)	88.18%
PBMDA (You et al., 2017)	91.72%
LLCMDA (Qu et al., 2018)	91.90%
EDTMDA (Chen et al., 2019)	91.92%
GBDT-LR (Zhou et al., 2020)	92.74%
MCLPMDA (Yu et al., 2019)	93.20%
GAEMDA (Li et al., 2021b)	93.56%
MTCL-MDA	94.56%

**Figure 3:**
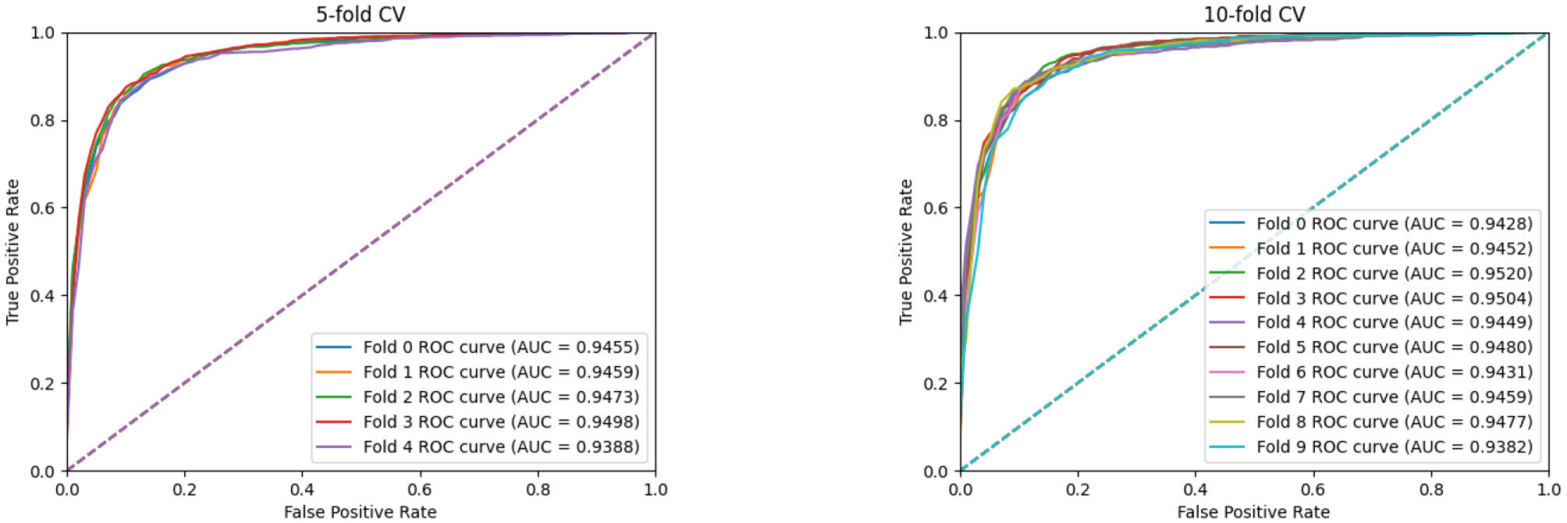
AUC curves of MTCL-MDA on 5(10)-fold cross-validation.

Furthermore, we conduct the 5-fold cross-validation experiments and the experimental results, are shown in Figure 3 and Table 2. It can be seen that all indicators can reflect the excellent effect of our model. We also run 10-fold cross-validation experiments and the *AUC* curves under the 10-fold cross-validation experiments, which indicates that the effect of the proposed MTCL-MDA is still stable and less affected by data splitting.

**Table 2:** Comparison of 5-fold cross-validation results between MTCL-MDA and GAEMDA model on HMDD v2.0 (%).

**Models**	**Testing set**	**AUC**	**ACC**	**PRE**	**REC**	**F1-score**
GAEMDA	1	93.21	84.30	80.43	90.35	85.10
	2	93.59	85.36	80.96	92.10	86.17
	3	94.34	86.23	84.74	88.41	86.54
	4	93.57	85.27	81.94	91.25	86.35
	5	93.07	83.47	78.77	91.39	84.61
	Average	93.56	84.93	81.37	90.70	85.75
MTCL-MDA	1	94.46	87.38	85.43	90.15	87.72
	2	94.56	88.17	85.10	92.54	88.66
	3	94.76	88.17	85.89	91.34	88.53
	4	94.95	87.62	85.12	91.16	88.04
	5	94.02	87.15	85.00	90.24	87.54
	Average	94.56	87.70	85.31	91.09	88.10

In order to provide additional evidence of our model's performance, we perform a detailed comparison with GAEMDA (Li et al., 2021b), which is the most advanced existing method in the field using an autoencoder to calculate the similarity of homogeneous nodes. Then feed the node embedding of miRNA and disease into the bilinear decoder to predict the potential correlations between miRNA and disease. However, the sparseness of node neighborhoods widely exists in various graphs, and the miRNA-disease bipartite graph is no exception. The GAEMDA (Li et al., 2021b) model does not deliberately consider this problem, while our method is exactly the opposite. And we integrated the contrastive learning strategy of miRNA (or disease) nodes based on topological neighbors and semantic neighbors, which can alleviate this problem. From the results in Table 2, we can see that the average of all metrics of our model is the best. Among them, the values of *AUC*, *ACC*, *PRE*, *REC*, and *F*1−*score* increased by 1, 2.97, 3.97, 0.69, and 1.14%, respectively.

### 3.3 Ablation experiment

We conduct the ablation experiments to evaluate the importance of topology-based and semantic-based contrastive learning modules. Table 3 shows the results of the ablation experiments. In Table 3, “w/o TCL” means the model removes the topology-based contrastive learning module, “w/o SCL” means the model removes the semantic-based contrastive learning module, “w/o TCL” means the model removes all contrastive learning module. The results show that the model achieved the worst performance when it did not use the contrastive learning module. The performance of the model is improved when it adopts topology-based or semantic-based contrastive learning modules. The best performance occurs when the model adopts both topology-based and semantic-based contrastive learning modules. At this time, the *AUC*, *ACC*, *PRE*, *REC*, and *F*1−*score* indicators obtained by the model increased by 1.27, 1.78, 2.49, 0.39, and 1.53% respectively. This proves that topology-based and semantic-based contrastive learning modules can work together and play a positive role in improving model performance.

**Table 3:** Results of ablation experiments of MTCL-MDA model on HMDD v2.0 (%).

**Models**	**AUC**	**ACC**	**PRE**	**REC**	**F1-score**
w/o CL	93.29	85.92	82.82	90.70	86.57
w/o TCL	94.00	86.26	84.73	88.87	86.92
w/o SCL	94.13	86.34	83.79	89.43	86.52
MTCL-MDA	94.56	87.70	85.31	91.09	88.10

### 3.4 Stability evaluation

**Figure 4:**
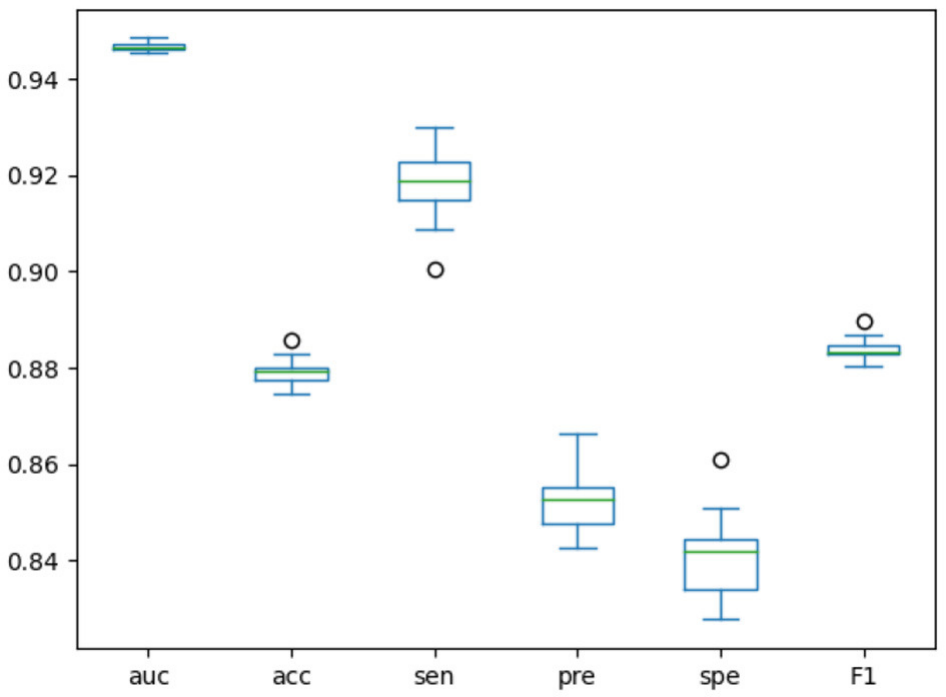
Boxplots of training model for 20 times.

We designed an experiment to make the model run randomly for 20 rounds, and drew the boxplots of the results of these 20 rounds, as shown in Figure 4. Boxplots can show how scattered a set of data is, detect and display outliers in the data, and clean them up. Based on these results, we could judge and observe the overall distribution of the data. When the data distribution is relatively concentrated, the boxes in the boxplot will be smaller, otherwise the boxes will be larger. When the midline is close to the upper edge of the boxplot, the data is concentrated in the upper half part, and when the midline is close to the bottom edge of the boxplot, the data is concentrated in the lower half part. The boxplot results of each indicator in Figure 4 further demonstrate the stability of our model.

In addition, we constructed parameter experiments to study the sensitivity of model performance to the involved hyperparameters. In the experiment, we kept the remaining parameters consistent and evaluated the impact of hyperparameters α, β_1_, β_2_, and β_3_ on model performance, as shown in Table 4. The results indicate that the performance of the model is not sensitive to the hyperparameters α, β_2_, and β_3_. Therefore we can set these three hyperparameters relatively easily. In addition, when the hyperparameter β_1_ is greater than 1e-6, the performance of the model decreases significantly; when β_1_ is less than 1e-6, the performance of the model improves significantly and tends to be stable. We also found that when the three parameters β_1_, β_2_, and β_3_ are all set to smaller numbers, the model performance is relatively stable. Therefore, we can set the hyperparameters β_1_, β_2_, and β_3_ to a smaller number, such as 1e-6.

**Table 4:** Results of parameter experiments of MTCL-MDA model on HMDD v2.0 (%).

**Parameters**	**Rounds**	**AUC**	**ACC**	**PRE**	**REC**	**F1-score**
	α = 0.1	93.56	87.13	84.26	86.56	88.85
β_1_ = 1e-6	α = 0.2	93.91	87.19	84.99	89.23	86.84
β_2_ = 1e-8	α = 0.5	94.15	88.24	83.58	91.07	88.32
β_3_ = 1e-6	α = 1.0	94.31	89.02	84.06	90.98	89.02
	α = 2.0	94.18	88.06	83.73	89.23	87.97
	β_1_ = 1e-4	91.13	83.46	81.74	87.48	86.60
α = 1.0	β_1_ = 1e-5	91.88	82.41	81.58	83.79	85.16
β_2_ = 1e-8	β_1_ = 1e-6	94.31	89.02	84.06	90.98	89.02
β_3_ = 1e-6	β_1_ = 1e-7	94.29	87.95	85.88	89.04	88.96
	β_1_ = 1e-8	94.04	87.26	85.01	87.29	88.15
	β_2_ = 1e-6	94.24	87.61	84.14	89.96	87.55
α = 1.0	β_2_ = 1e-7	94.12	87.42	84.26	89.41	87.33
β_1_ = 1e-6	β_2_ = 1e-8	94.31	89.02	84.06	90.98	89.02
β_3_ = 1e-6	β_2_ = 1e-9	94.19	87.75	84.09	90.33	87.71
	β_2_ = 1e-1	94.26	87.42	84.57	90.24	87.41
	β_3_ = 1e-4	94.27	87.47	84.04	89.78	87.40
α = 1.0	β_3_ = 1e-5	94.23	87.75	84.47	89.87	87.66
β_1_ = 1e-6	β_3_ = 1e-6	94.31	89.02	84.06	90.98	89.02
β_2_ = 1e-8	β_3_ = 1e-7	94.35	87.84	84.58	91.16	87.87
	β_3_ = 1e-8	94.28	87.79	84.56	91.07	87.82

### 3.5 Case study

To validate the predictive performance of our proposed model MTCL-MDA in practical scenarios, we perform case studies and Table 5 presents the corresponding results. The known biological experiment results show that there is some relationship between miRNA and cardiomyopathy. Cardiomyopathy is a relatively serious heart disease. Once patients have symptoms, it will affect their normal life and work. As the disease progresses, the symptoms of heart failure will further aggravate, and symptoms such as edema and dyspnea will appear. The sick will not be able to live and rest normally, and the quality of life will further decline, which will bring heavy burden and pain to the family and society. Patients with hypertrophic cardiomyopathy and arrhythmogenic cardiomyopathy, especially young people, are even at risk of sudden death. Therefore, we selected cardiomyopathy to predict its associated miRNAs.

**Table 5:** Top 20 cardiomyopathy-related miRNAs predicted by MTCL-MDA based on HMDD v2.0.

**miRNA**	**miR2Diseas**	**miRNA**	**miR2Diseas**
hsa-mir-27a	Definited	hsa-mir-181b	Undefinited
hsa-mir-499a	Undefinited	hsa-mir-195	Definited
hsa-mir-150	Undefinited	hsa-mir-125b	Definited
hsa-mir-21	Definited	hsa-mir-199b	Definited
hsa-mir-1	Definited	hsa-mir-27b	Definited
hsa-mir-23a	Definited	hsa-mir-214	Definited
hsa-mir-199a	Definited	hsa-mir-23b	Definited
hsa-mir-196a	Undefinited	hsa-mir-155	Definited
hsa-mir-126	Undefinited	hsa-mir-9	Undefinited
hsa-let-7i	Undefinited	hsa-mir-133a	Definited

In case studies, we execute experiments with the following steps: when training the model, firstly, delete the association information between cardiomyopathy (including dilated cardiomyopathy and hypertrophic cardiomyopathy) and miRNA from the 5,430 MDAs, and secondly, train the model by randomly collecting corresponding negative samples, and next use the association between cardiomyopathy (including dilated cardiomyopathy and hypertrophic cardiomyopathy) and all miRNAs as test samples. Then output the top 10 miRNAs which are predicted associated with cardiomyopathy. After comparing with the real labels of known samples, it is known that the prediction results are completely correct. This further verifies the superiority of our model. Our model MTCL-MDA has practical implications in the field of mining disease-related miRNAs and providing reliable guidance for disease treatment.

In biological experiments, it has been proved that miRNA is related to various diseases, such as hsa-mir-29a is related to various tumor diseases, including but not limited to breast tumor, liver tumor, gastric tumor, and so on. It is likely to be involved in more diseases, so we design experiments to explore whether hsa-mir-29a is interrelated other diseases. The association data between hsa-mir-29a and disease is removed when training the model, and the corresponding negative samples are selected randomly. Finally, we take the association of hsa-mir-29a with all diseases as test samples and output the predicted association probabilities. The predicted results are shown in Table 6, which manifests that miRNAs are not only associated with various tumor diseases, but also involved in Alzheimer's disease (Li et al., 2022a), Parkinson's disease and other mental diseases, some of which have also been verified in the latest database.

**Table 6:** Predicted diseases associated with hsa-mir-29a based on MTCL-MDA (TOP 20).

**Disease**	**dbDEMC**	**miR2Diseas**
Breast neoplasms	Definited	Undefinited
Carcinoma, hepatocellular	Definited	Undefinited
Stomach neoplasms	Definited	Undefinited
Carcinoma, hepatocellular	Definited	Undefinited
Ovarian neoplasms	Definited	Undefinited
Mouth neoplasms	Definited	Definited
Parkinson disease	Definited	Undefinited
Colonic neoplasms	Definited	Definited
Crohn disease	Undefinited	Undefinited
Stomach neoplasms	Definited	Undefinited
Heart failure	Undefinited	Undefinited
Schizophrenia	Undefinited	Undefinited
Colorectal neoplasms	Definited	Undefinited
Tuberculosis, pulmonary	Undefinited	Undefinited
Aortic aneurysm, thoracic	Definited	Undefinited
Myocardium	Undefinited	Definited
Melanoma	Definited	Undefinited
Biliary atresia	Undefinited	Undefinited
Liver cirrhosis	Undefinited	Undefinited
Endomyocardial fibrosis	Undefinited	Undefinited

## 4 Conclusion

This study propose a model named MTCL-MDA based on graph collaborative filtering, which can accurately predict MDAs. To fully utilize the topological information, we present a contrastive learning strategy based on topological neighborhood. And we designed a contrastive learning strategy based on semantic neighborhood to alleviate the problem of the noise information introduced by the topological contrastive learning strategy and the sparse topological neighborhood. The comparison results with the current methods fully demonstrate the high-performance of the MTCL-MDA model. Furthermore, the case analysis suggests that the MTCL-MDA model can be an option for the discovery of disease-associated miRNAs and miRNA-associated diseases, thereby providing constructive suggestions for disease treatment and diagnosis. Since the proposed model uses a lightweight graph collaborative filtering model as the encoder, this may result in the loss of some information during message propagation. In addition, the model will involve relatively complex operations when calculating the contrastive learning loss. In future work, we will focus on solving these problems and improving the applicability of the model.

## Data availability statement

The datasets presented in this study can be found in online repositories. The data and code are publicly available at: https://github.com/Lqingquan/MTCL-MDA.

## Author contributions

LZ: Writing – review & editing, Validation. QL: Writing – original draft, Methodology. XF: Writing – review & editing. HC: Writing – review & editing.
