# Supplementary material for: An efficient model for predicting human diseases through miRNA based on multiple-types of contrastive learning
Source: Front Microbiol. 2023 Dec 14;14:1325001. doi: 10.3389/fmicb.2023.1325001 (PMC10755968; doi:10.3389/fmicb.2023.1325001)
Supplement: Supplementary file 1 [file Data_Sheet_1.ZIP › MTCL-MDA/frontiers-highlighted.pdf]

An efficient model for predicting human diseases through miRNA based on multiple-types of contrastive learning

Qingquan Liao [1], Xiangzheng Fu [1], Linlin Zhuo [2,*], and Hao Chen [1,*]

[1] *College of Computer Science and Electronic Engineering, Hunan University, Changsha, China*
[2] *School of Data Science and Artificial Intelligence, Wenzhou University of Technology, Wenzhou, China*

Correspondence*:
Linlin Zhuo and Hao Chen
email: zhuoninnin@163.com, chenhao@hnu.edu.cn

ABSTRACT

Multiple studies have demonstrated that microRNA (miRNA) can be deeply involved in the regulatory mechanism of human microbiota, thereby inducing disease. Developing effective methods to infer potential associations between microRNAs (miRNAs) and diseases can aid early diagnosis and treatment. Recent methods utilize machine learning or deep learning to predict miRNA-disease associations (MDAs), achieving state-of-the-art performance. However, the problem of sparse neighborhoods of nodes due to lack of data has not been well solved. To this end, we propose a new model named MTCL-MDA, which integrates multiple-types of contrastive learning strategies into a graph collaborative filtering model to predict potential MDAs. The model adopts a contrastive learning strategy based on topology, which alleviates the damage to model performance caused by sparse neighborhoods. In addition, the model also adopts a semantic-based contrastive learning strategy, which not only reduces the impact of noise introduced by topology-based contrastive learning, but also enhances the semantic information of nodes. Experimental results show that our model outperforms existing models on all evaluation metrics. Case analysis shows that our model can more accurately identify potential MDA, which is of great significance for the screening and diagnosis of real-life diseases. Our data and code are publicly available at: https://github.com/Lqingquan/MTCL-MDA.

Keywords: contrastive Learning, diagnosis and treatment, graph collaborative filtering, human microbiota, miRNA-disease associations, sparse neighborhoods.

1 INTRODUCTION

MicroRNA(miRNA) is a kind of RNA molecule that is single-stranded and generally consists of 19-25 nucleotides (Cuperus et al., 2011), which is endogenous non-protein-coding, and highly conserved in evolution (Ambros, 2004). Relevant studies have shown that miRNA is involved in the human intestinal microbial environment, thereby affecting the pathogenesis of certain intestinal inflammation (Friedman et al., 2009). Some miRNAs bind to mRNA, thereby inhibiting activities such as mRNA degradation, causing the downregulation of mRNA expression function (Gebert and MacRae, 2019). Generally, miRNA exists in human peripheral blood, but it may also appear in intestinal fluid, saliva and other body fluids (Weber et al., 2010). Recent exploration has found that miRNA serves as a mediator to guide the interaction

between cells and microbiota (Ji et al., 2018). In addition, certain metabolites produced by microorganisms can affect the expression of miRNA, thereby affecting the host's microecology. Some studies have verified that abnormal function of miRNA is involved in the pathogenesis of certain diseases (Park et al., 2017). Related studies have also revealed that miRNA and microbial communities are related to Inflammatory bowel disease (James et al., 2020; Qin et al., 2010).

MiRNA regulates the flow and expression of genetic information in space and time through post-transcriptional gene regulation or silencing (Zhang et al., 2018), which involves approximately 30 to 90 human genes (Cai et al., 2009). The ability of miRNAs to regulate apoptosis and growth of cells has been demonstrated by numerous studies (Neilson et al., 2007). Tumor formation often results when cells display abnormal growth and loss of apoptotic function (Hill and Tran, 2021). Although it is not completely clear how miRNAs regulate the development and maturation of nervous system and their physiological functions, it has been confirmed that the expression of miRNAs in nervous system is characterized by high time sequence, high conservation and high specificity (Cao et al., 2016). At present, it is widely believed among researchers that miRNAs perform specific regulatory functions in the nervous system's development and operation. Many studies indicate that miRNAs are intricately involved in the precise regulation for the function and development of nervous system (Christensen and Schratt, 2009). Once the regulation is chaotic, it will inevitably lead to disease. Although the correlation between miRNAs and cerebrovascular diseases is still being explored, according to the analysis of relevant research data, miRNAs may also contribute to the development of cerebrovascular diseases (Hu et al., 2015). On one hand, miRNA can be used as a therapeutic target (Ganju et al., 2017) to achieve gene regulation (Weiland et al., 2012), and on the other hand, it can serve as a biomarker of disease diagnosis and disease screening (Mo et al., 2012).

The traditional methods for detecting miRNA mainly include Northern Analysis (Válóczi et al., 2004), Microarray (Li and Ruan, 2009) and Quantitative Real-time PCR (Benes and Castoldi, 2010). Northern Analysis (Válóczi et al., 2004) is a common method for detecting RNA based on hybridization (Várallyay et al., 2008), and it is one of the earliest methods for miRNA analysis. This method is simple and feasible, and most laboratories can operate without additional capital investment and equipment update. However, the analysis process requires a lot of manual operations, is not suitable for large-scale screening experiments, and it is usually unable to effectively distinguish miRNAs with small sequence differences. Microarray (Li and Ruan, 2009) also detects miRNA based on hybridization principle, which analyzes and understands the mechanism of miRNA expression regulation and gene expression regulated by miRNA by measuring the expression level of miRNA in a specific process. However, this method requires sufficient initial RNA samples, and it is difficult to clearly distinguish miRNAs with small differences, as well as precursor miRNAs with the same sequence and mature active miRNAs. Real-time quantitative PCR is a method that completes the overall analysis process by adding a fluorescent group to the DNA amplification reaction (Benes and Castoldi, 2010). However, it is of heavy workload and high cost. Through the implementation of reliable methodologies like these, researchers have established extensive bioinformatics databases that house experimentally verified miRNAs and their associations with diseases. These databases are widely acknowledged as dependable sources of information. For example, dbDEMC is a database of miRNAs related to human cancer (Xu et al., 2022). HMDD is a database of miRNA-related diseases (Huang et al., 2019). MiR2Disease is a miRNA-related disease database developed by Harbin University of Technology (Jiang et al., 2009). They provide a lot of valuable data support for future research.

The researches have proposed many computational methods to predict the MDAs well. Most of these approaches assume that miRNAs which have similar functions are potentially associated with diseases having similar phenotypes. For example, Chen applies regularization and semi-supervised strategies to

predict miRNA-disease correlations (Chen and Yan, 2014). Jiang proposes the network-based method to infer the potential MDAs (Jiang et al., 2010). Luo systematically prioritizes disease-associated miRNAs using transduction learning-based collective prediction. Zhang et al. use a fast network link reasoning method based on linear neighborhood similarity. First, the known miRNA disease association is expressed as a binary network, and the miRNA is expressed as a correlation spectrum, so is the disease. Then the fast linear neighborhood similarity measure and the correlation curve are proposed to estimate the MDAs (Zeng et al., 2016). Huang integrates the nuclear similarity of miRNA-disease Gaussian interaction profiles into the original multiple data, and proposes a novel prediction model called PBMDA (You et al., 2017). By integrating multiple data, Chen et al. further propose an induction matrix strategy to forecast MDAs (Li and Ruan, 2009; Benes and Castoldi, 2010).

Recently, machine learning and deep learning have been leveraged in the field of biology, such as prediction of gene regulatory (Peng et al., 2022; Gao et al., 2023; Wang et al., 2022), drug discovery (Li et al., 2021a), ncRNA–protein interactions (Liu et al., 2022). Biomedical scientists are drawing inspiration from this approach, and utilizing machine learning algorithms to forecast possible correlations between miRNAs (microRNAs) and diseases, resulting in enhanced accuracy of prediction outcomes. Zhang et al. propose a new method which predictes MDAs using automatic encoding machines and extractes features based on unsupervised rule (Zhang et al., 2019). Fu presentes a deep learning ensemble model, named DeepMDA, that leverages stacked autoencoders to extract complex features from similarity data. Ding developes a deep learning model, known as VGAE-MDA, which is based on variational autoencoding of graphs and is intended for forecasting MDAs (Ding et al., 2021).

Due to the great progress of graph neural network (GNN)s on graph-structured data (Cai et al., 2021; Wang et al., 2023, 2022), GNN-based models have been developed to forecast miRNA-disease correlations. Wang et al. estimate between miRNAs-diseases correlations using a hybrid model that combines graph convolutional networks and convolutional neural networks, which are boosted by multi-channel attention (Wang and Chen, 2023). Li et al. suggest a model named GAEMDA, that utilizes autoencoder-based GNNs to recognize miRNA-disease correlations (Li et al., 2021b). Moreover, Li et al. integrate attention mechanism into a hierarchical GNN to predict miRNA-disease correlations (Li et al., 2022b).

The above methods have proven their success, but they do not fully consider the sparseness of node neighborhoods, including node topological neighborhood sparseness and node semantic neighborhood sparseness that degrade the performance of these models. In this study, we propose a graph collaborative filtering based model that integrates a contrastive learning strategy for topological neighbors of miRNA (or disease) nodes and a contrastive learning strategy for semantic neighbors of nodes. Our model can well alleviate the node sparse neighborhood problem and accurately predict miRNA-disease correlations. Experimental results prove the superiority of our model to predict the association between miRNAs and diseases. Our contributions are summarized as:

1. We propose a novel method to predict miRNA-disease associations, integrating a contrastive learning strategy into a graph collaborative filtering.
2. We propose a contrastive learning strategy based on the topological neighbors of miRNA (or disease) nodes, which can be used to fully mine the topological information in the miRNA-disease bipartite graph.
3. The semantic similarity between miRNA and disease is used to enrich the neighborhood information of nodes in the miRNA-disease bipartite graph, and a contrastive learning strategy for node semantic neighbors is proposed. This reduces the noise impact brought by the contrastive learning strategy of

node topological neighbors, while alleviating the problem of sparse neighborhoods of nodes in the dataset.

4. Based on the MDAs datasets, we have constructed multiple sets of comparative experiments to evaluate the effectiveness and stability of our model. And the corresponding case analysis proves that the MTCL-MDA model can provide a certain degree of advice for early intervention in diseases.

2 MATERIALS AND METHODS

Based on the graph collaborative filtering model, we apply contrastive learning strategies to node topological neighbors and node-based semantic neighbors, respectively, and propose a model named MTCL-MDA. In general, it is easy to aggregate topological neighbors in GNN-based research. However, for semantic-based neighbors, although various methods have been tried, the results are not satisfactory. And when the topological neighborhood of nodes is sparse, semantic information (such as feature vectors) becomes more critical for the accurate representation of nodes. Therefore, the importance of semantic neighbors is self-evident. In our study, contrastive learning strategies are applied in two aspects to alleviate this problem, and the final experimental results are also very satisfactory. We present our approach in the following sections.

2.1 Problem formulation

Our main goal is to predict unknown MDAs based on observed MDAs. These known MDAs can be used as the basis for constructing a bipartite graph G = (M, D, A). M represents miRNAs collection, D represents disease collection, and A represents MDAs.

Our main goal is to predict unknown MDAs based on observed MDAs. These known MDAs can be used as the basis for constructing a bipartite graph $G = (M, D, E)$. $M = \{m_1, ..., m_N\}$ represents miRNAs collection, $D = \{d_1, ..., d_N\}$ represents disease collection, and A represents MDAs. If there is a link from node m_i to d_j, $A_{ij} = 1$, otherwise $A_{ij} = 0$.

Predicting MDAs involves node neighborhood information. $N^h(v_i)$ represents the set of neighbors of node v_i within h-order. In our research, we focus on the unweighted graph, v_i is regarded as the target node and $v_j \in N^h(v_i)$ is the neighbor within the h-order.

Link prediction problems (Lü and Zhou, 2011) are divided into time link prediction (predicting potential new links in evolutionary networks) and topological link prediction (inferring unknown links in static networks). Similar to the latter, given partial observations of the topology, it is expected to predict unknown links. In practical problems, given the correlation topological structure of some observed miRNAs and diseases, we can predict the unknown correlation, that is, whether the miRNA is related to the disease.

2.2 Model architecture

Our model mainly consists of three parts: graph collaborative filtering, contrastive learning of miRNA (or disease) nodes, and MDAs prediction. The main components of the model are shown in Figure 1. We apply graph collaborative filtering to disseminate network information in the miRNA-disease bipartite graph, and update node embeddings by aggregating neighbors. And the model integrates the contrastive learning strategy of topological neighbors and the contrastive learning strategy of semantic neighbors. Immediately, it can not only reduce the negative impact of noise caused by purely using topology contrastive learning strategy, but also alleviate the problem of sparse topological neighborhood of nodes. Finally, we concatenate

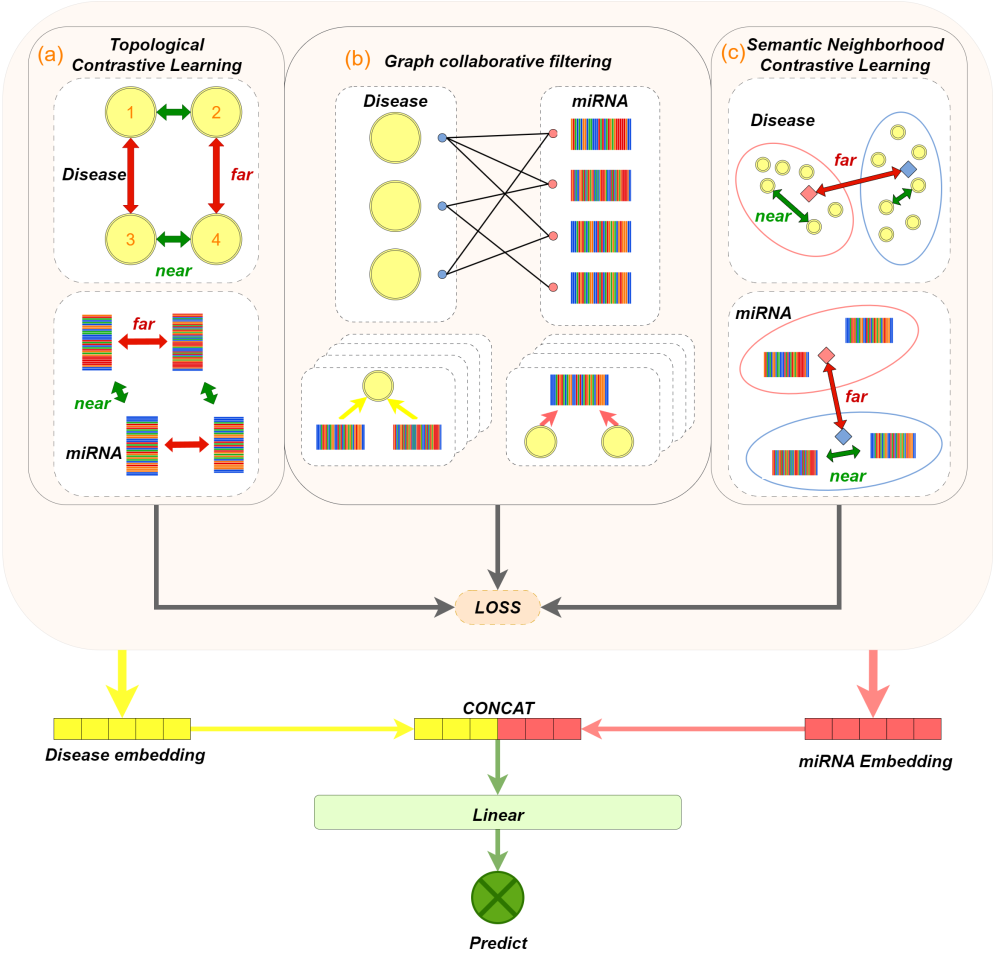

Figure 1. MTCL-MDA model architecture figure. (a) The dashed box on the left shows the contrastive learning of disease nodes based on topological neighborhood. For a disease node 1, its embedding and the embedding obtained in the even layer of GNN constitute a positive sample; its embedding in the even layer constitutes a negative pair with the embedding of other disease nodes not connected to it in the even layer. Conduct contrastive learning training so that the distance between nodes with similar structure (such as 1 and 2, 3 and 4) is smaller, and the distance between 1 (2) and 3 (4) with dissimilar structure is larger. (b) Brief process of representative graph collaborative filtering, where four layers of aggregation operations are performed. (c) The dashed box on the left represents the contrastive learning of disease nodes based on semantic neighborhood. The orange and blue solid squares represent the cluster centers, the nodes in the cluster and the cluster centers constitute positive samples, and the nodes in the cluster and other cluster centers constitute negative samples. In this way, contrastive learning training is carried out, so that the distance between nodes with similar feature vectors is smaller, and the distance between nodes with dissimilar feature vectors is larger. Processing on miRNA nodes is similar to disease nodes.

the output miRNA and disease node embeddings to obtain a paired vector, and then feed it into MLPs to determine whether there exists a association between miRNA and disease.

2.3 Graph collaborative filtering

As mentioned at the beginning of this section, GNN-based approaches generate miRNA (or disease) embeddings by applying propagation and prediction functions to the topology composed of MDAs. In this study, a graph collaborative filtering model is employed to complete the propagation process. Specifically,

Our propagation function is as follows:

$$X_m^{l+1} = \sum_{d \in N_m} \frac{1}{\sqrt{|N_m|\,|N_d|}} X_d^l \tag{1}$$

$$X_d^{l+1} = \sum_{d \in N_d} \frac{1}{\sqrt{|N_d|\,|N_m|}} X_m^l, \tag{2}$$

where X_m^l and X_d^l denote the embeddings of miRNA and disease nodes on l_{th}-layer network, respectively. N_m and N_d denote the neighbor sets of nodes m and d, respectively. After the L layer propagation, We utilize the weighted sum function as the readout function and aggregate the representations from all layers to derive the ultimate representation, as depicted below:

$$X_m = \frac{1}{L+1} \sum_{l=0}^{L} X_m^k, \tag{3}$$

$$X_d = \frac{1}{L+1} \sum_{l=0}^{L} X_d^k, \tag{4}$$

where X_m and X_d respectively represent the final node embedding of nodes m and d. And then calculate the inner product between nodes m and d as the correlation score:

$$\widehat{y}_{m,d} = \mathbf{X_m}^T X_d, \tag{5}$$

where $\widehat{y}_{m,d}$ represents the predicted score of (m, d) pair. The BPR loss function (Rendle et al., 2012) is employed to train direct MDAs. Specifically, the loss function can make positive MDAs scores tend to be larger than negative association scores. Formally, the objective function of BPR loss is as follows:

$$L_{BPR} = \sum_{(m,i,j) \in O} -\log \sigma(\widehat{y}_{m,i} - \widehat{y}_{m,j}), \tag{6}$$

where L_{BPR} and σ represent BPR loss and $sigmod$ activation, respectively. $O = \{(m, i, j)|R_{m,i} = 1, R_{m,j} = 0\}$ represents the paired training data, and j indicates the sampled diseases with which miRNA m is not associated. By optimizing L_{BPR}, our proposed model can model these observed MDAs. However, the higher-order neighbors within miRNA (or disease) is also valuable for prediction task. For example, miRNAs within the same cluster have a high probability of being linked to the same miRNAs (or diseases). Next, we demonstrate two contrastive learning strategies to capture the respective latent neighbor relationships of miRNAs and disease nodes.

2.4 Contrastive learning strategy based on topological neighborhood

This subsection will demonstrate a contrastive learning strategy based on miRNA (or disease) nodes. More specifically, each miRNA (or disease) node is learned contrastively with its own topological neighbors. Easily, the node embeddings of these neighbors can be aggregated by GNN propagation. Bipartite graphs are formed on direct MDAs, and even layer propagation naturally gathers information from homogeneously structured neighbors. For example, node representations of similar neighbors can be obtained from the output of even-numbered layers (such as 2, 4, 6) based on GNN models. We regard the embedding of the

miRNA node itself and its corresponding output embedding in even layers as positive samples. Based on the InfoNCE (Aitchison, 2021) loss function, we propose a structure-contrastive learning objective as follows:

$$\mathcal{L}_S^M = \sum_{m \in M} -\log \frac{\exp\left(\left(\mathbf{X}_m^{(k)} \cdot \mathbf{X}_u^{(0)}/\tau\right)\right)}{\sum_{d \in M} \exp\left(\left(\mathbf{X}_m^{(k)} \cdot \mathbf{X}_d^{(0)}/\tau\right)\right)}. \tag{7}$$

M means the set of miRNA nodes. $X_m^{(k)}$ is the normalized output of k_{th} layer in GNN, k is an even number, and τ is the temperature hyperparameter on the numerator and denominator in the softmax function. Similarly, the topological neighbor contrastive learning strategy for disease nodes can be summarized as follows:

$$\mathcal{L}_S^D = \sum_{d \in D} -\log \frac{\exp\left(\left(\mathbf{X}_d^{(k)} \cdot \mathbf{X}_u^{(0)}/\tau\right)\right)}{\sum_{m \in D} \exp\left(\left(\mathbf{X}_d^{(k)} \cdot \mathbf{X}_d^{(0)}/\tau\right)\right)}. \tag{8}$$

where D means the set of disease nodes.

Integrating the topological neighbor contrastive learning strategy of miRNA nodes and the topological neighbor comparison learning strategy of disease nodes, the following can be obtained:

$$\mathcal{L}_S = \mathcal{L}_S^M + \alpha \mathcal{L}_S^D. \tag{9}$$

where α represents the adjustment parameter for two losses.

2.5 Contrastive learning strategy based on semantic neighborhood

As mentioned in Section 2.4, the topological contrastive learning strategy focuses on the neighbors defined by the miRNA-disease bipartite graph. However, it only takes into account the loss of contrast between a miRNA (or disease) node and its homogeneous neighbors. Meanwhile, it indiscriminately computes the contrastive loss of miRNA (or disease) nodes, which will inevitably introduce noise information. To mitigate suffering from topological neighbor noise, we consider extending the contrastive learning strategy by incorporating miRNA (or disease) node semantic neighbors. For a miRNA (or disease) node, its semantic neighbors refer to nodes that are unreachable on the miRNA-disease bipartite graph but have similar feature vectors.

Motivated by previous work (Mirman, 2011), we can determine semantic neighbors by learning latent prototypes of nodes. Therefore, we construct a prototype-based contrastive learning objective to identify potential semantic neighbors of miRNA (or disease) nodes. Meanwhile, the semantic neighbor-based contrastive learning strategy is integrated into the whole contrastive learning framework to better capture the semantic features of miRNAs(or diseases). Specifically, miRNA (or disease) nodes with similar node embeddings will be assigned into the same clusters using a clustering algorithm. These clusters are represented by the central nodes of the clusters, which are called prototypes. The process can use the EM learning algorithm (Kushary, 1998) to optimize the proposed prototype-contrastive learning function. Optimized by maximizing the log-likelihood of the following probability distribution function:

$$\sum_{m \in \mathcal{M}} \log p\left(\mathbf{e}_m \mid \Theta, \mathbf{R}\right) = \sum_{m \in \mathcal{M}} \log \sum_{\mathbf{c}_i \in C} p\left(\mathbf{e}_m, \mathbf{c}_i \mid \Theta, \mathbf{R}\right) \tag{10}$$

And Θ indicates all parameters, $\mathbf{R}$ indicates the miRNA-disease bipartite graph, and c_i is the potential comparison prototype of the miRNA node m. Similarly, we can define an optimization objective function for the set of diseased nodes:

$$\sum_{d \in \mathcal{D}} \log p\left(\mathbf{e}_d \mid \Theta, \mathbf{R}\right) = \sum_{d \in \mathcal{D}} \log \sum_{\mathbf{c}_i \in C} p\left(\mathbf{e}_d, \mathbf{c}_i \mid \Theta, \mathbf{R}\right) \tag{11}$$

Applying the InfoNCE (Aitchison, 2021) loss function, we can optimize the following objectives based on contrastive strategies:

$$\mathcal{L}_P^D = \sum_{m \in M} -\log \frac{\exp\left(e_m \cdot c_i / \tau\right)}{\sum_{c_t \in C} \exp\left(e_m \cdot c_t / \tau\right)}, \tag{12}$$

where c_i is the prototype node of miRNA node m, which is obtained by clustering all miRNA node embeddings by k-means algorithm. The set of miRNA nodes can be assigned into k clusters, and the value of k can be set as required. A similar process also applies to disease node sets:

$$\mathcal{L}_P^D = \sum_{d \in D} -\log \frac{\exp\left(e_d \cdot c_j / \tau\right)}{\sum_{c_t \in C} \exp\left(e_d \cdot c_t / \tau\right)}, \tag{13}$$

where c_j represents the prototype node of the disease node d. By integrating the contrastive learning process of miRNA node sets and the contrastive learning process of miRNA node sets, we can get the final semantic neighbor-based optimization objective:

$$\mathcal{L}_P = \mathcal{L}_P^M + \alpha \mathcal{L}_P^D \tag{14}$$

According to this semantic neighbor-based contrastive learning strategy, on the one hand, it can reduce the impact of noise brought about by topology contrastive learning, and on the other hand, it can alleviate the problem of sparse node neighborhoods.

Therefore, the overall loss of the model is:

$$\mathcal{L} = \mathcal{L}_{BPR} + \beta_1 \mathcal{L}_S + \beta_2 \mathcal{L}_P + \beta_3 \left|\left|\Theta\right|\right|_2 \tag{15}$$

where β_1, β_2 and β_3 are the parameter to control the weight, and Θ denotes all parameters of GNN model. We can apply EM algorithm to optimize the solution.

2.6 Prediction of MDAs based on MLP

We integrated the obtained embeddings of miRNA and disease nodes to further predict whether they are positive pairs or negative pairs. Commonly used integration methods include Hadamard product, vector inner product, vector addition, and concatenate operations. In this study, we empirically selected the concatenate operation:

$$\widehat{y}_{m,d} = concatenate(\mathbf{X_m}, \mathbf{X_d}), \tag{16}$$

where X_m and X_d represent the embeddings of miRNA m and disease d, respectively. If the miRNA m is associated with the disease d, then the (m, d) pair is positive, otherwise it is negative. The concatenated

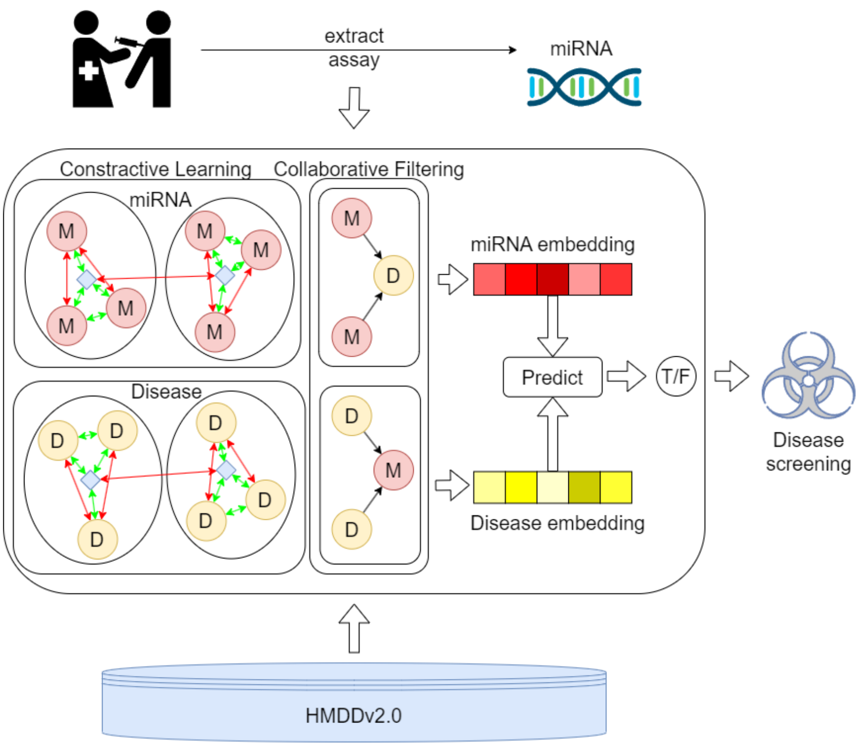

Figure 2. The process of the proposed MTCL-MDA model for preliminary disease screening.

embedding representation x will be fed into MLPs, and finally output:

$$S(x) = \frac{1}{1 + \exp(-x)}. \tag{17}$$

Then the BCE loss (Wu et al., 2020) for the classification is calculated by:

$$loss = -y \log(S(\widehat{y}_{m,d})) - (1 - y)\log(1 - S(\widehat{y}_{m,d})), \tag{18}$$

where y represents the true MDA in the dataset, and its value is 0 or 1. $S(\widehat{y}_{m,d})$ represents the label predicted by the classifier.

2.7 Preliminary disease screening

Figure 2 presents the process of the proposed MTCL-MDA model for preliminary disease screening. First, samples are taken from the patient's relevant organs or tissues and assayed to extract key miRNA components. Then, the proposed model was used to predict diseases associated with this miRNA in the HMDD v2.0 database. The first process involves more complex biochemical testing and analysis. The proposed model can serve as a preliminary screening tool for the disease and play an active role in the second process. And our webserver is publicly accessible at: https://huggingface.co/spaces/ZZCrazy00/MDA.

3 RESULTS

We use pytorch tool for building deep learning frameworks, to implement the MTCL-MDA model. To assess the effectiveness of the proposed model, we conducted extensive comparative experiments using the miRNA-disease dataset. This section mainly includes the following parts.

Table 1. Comparison of MTCL-MDA model with other advanced methods on AUC indicator.

Models	AUC
WBSMDA (Chen et al., 2016)	81.85%
RFMDA (Chen et al., 2018)	88.18%
PBMDA (You et al., 2017)	91.72%
LLCMDA (Qu et al., 2018)	91.90%
EDTMDA (Chen et al., 2019)	91.92%
GBDT-LR (Zhou et al., 2020)	92.74%
MCLPMDA (Yu et al., 2019)	93.20%
GAEMDA (Li et al., 2021b)	93.56%
MTCL-MDA	94.56%

3.1 Datasets and experimental settings

We use datasets downloaded from HMDD v2.0 database. It contains 495 miRNAs, 383 diseases and 5430 MDAs verified by experiments. The association information is represented by a matrix A with the size of (495, 383), where $A_{ij} = 1$ indicates that the i_{th} miRNA associated with the j_{th} diseaseand $A_{ij} = 0$ indicates that there is no association.

We evaluate the performance of the proposed model in terms of AUC, $Accuracy$ (abbreviated as ACC), $Specificity$ (abbreviated as SPE), $Precision$ (abbreviated as PRE), $Recall$ (abbreviated as REC), $F1 - score$ and other metrics. The metrics used in our experiments are expressed as follows:

$$Acc = \frac{TP + TN}{TP + TN + FP + FN}, \quad Spe = \frac{TN}{TN + FP}, \tag{19}$$

$$Sen = \frac{TP}{TP + FN}, \quad Pre = \frac{TP}{TP + FP}, F1 - score = 2 * \frac{Pre * Sen}{Pre + Sen} \tag{20}$$

In the above equations, TP represents the positive association ratio of miRNA-disease correctly classified, FP represents the positive association ratio of miRNA-disease misclassified, TN represents the negative association ratio of miRNA-disease correctly classified, and FN represents misclassified miRNA-disease negative association ratio. And we compare the proposed model against the following eight baselines. WBSMDA (Chen et al., 2016) and RFMDA (Chen et al., 2018) integrates multiple similarity relations into a unified network to identify potential MDAs. PBMDA (You et al., 2017) integrates three interrelated networks and then predicted potential MDAs based on a depth-first search strategy. LLCMDA (Qu et al., 2018) is a method that utilizes locally constrained linear coding for predicting MDAs. EDTDA (Chen et al., 2019) is an innovative method that utilizes decision tree-based algorithms to predict MDAs. GBDT-LR (Zhou et al., 2020) combines gradient boosted decision trees and logistic regression to predict MDAs. MCLPMDA (Yu et al., 2019) predicts MDAs based on matrix completion. GAEMDA (Peng et al., 2022) predicts MDAs based on GNN and autoencoder.

3.2 Performance evaluation

We evaluate the performance of each model in terms of the metric AUC and the experimental results are reported in Table 1. Overall, all models achieved good results except the WBSMDA (Chen et al., 2016) model, which may be because most models have integrated and processed MDAs information. For example, the EDTMDA (Chen et al., 2019) and GBDT-LR (Zhou et al., 2020) models use an ensemble learning

Table 2. Comparison of 5-fold cross-validation results between MTCL-MDA and GAEMDA model on HMDD v2.0 (%).

models	Testing set	AUC	ACC	PRE	REC	F1-score
GAEMDA	1	93.21	84.30	80.43	90.35	85.10
	2	93.59	85.36	80.96	92.10	86.17
	3	94.34	86.23	84.74	88.41	86.54
	4	93.57	85.27	81.94	91.25	86.35
	5	93.07	83.47	78.77	91.39	84.61
	Average	93.56	84.93	81.37	90.70	85.75
MTCL-MDA	1	94.46	87.38	85.43	90.15	87.72
	2	94.56	88.17	85.10	92.54	88.66
	3	94.76	88.17	85.89	91.34	88.53
	4	94.95	87.62	85.12	91.16	88.04
	5	94.02	87.15	85.00	90.24	87.54
	Average	94.56	87.70	85.31	91.09	88.10

strategy, and the MCLPMDA (Yu et al., 2019) model uses a matrix completion strategy. We find that the GAEMDA (Li et al., 2021b) model achieves the second best results. The reason lies in it not only considers the topological information in the miRNA-disease bipartite graph in spite of fully considering the feature information of the nodes by using an autoencoder. It can also be seen from Table 1 that among all the models, the proposed MTCL-MDA performs best on AUC metric, and the reason is that the proposed MTCL-MDA considers not only the node topological information but also the topological neighborhood of nodes is sparse.

Furthermore, we conduct the 5-fold cross-validation experiments and the experimental results, are shown in Figure 3 and Table 2. It can be seen that all indicators can reflect the excellent effect of our model. We also run 10-fold cross-validation experiments and the AUC curves under the 10-fold cross-validation experiments, which indicates that the effect of the proposed MTCL-MDA is still stable and less affected by data splitting.

In order to provide additional evidence of our model's performance, we perform a detailed comparison with GAEMDA (Li et al., 2021b), which is the most advanced existing method in the field using an autoencoder to calculate the similarity of homogeneous nodes. Then feed the node embedding of miRNA and disease into the bilinear decoder to predict the potential correlations between miRNA and disease. However, the sparseness of node neighborhoods widely exists in various graphs, and the miRNA-disease bipartite graph is no exception. The GAEMDA (Li et al., 2021b) model does not deliberately consider this problem, while our method is exactly the opposite. And we integrated the contrastive learning strategy of miRNA (or disease) nodes based on topological neighbors and semantic neighbors, which can alleviate this problem. From the results in Table 2, we can see that the average of all metrics of our model is the best. Among them, the values of AUC, ACC, PRE, REC, and $F1 - score$ increased by 1%, 2.97%, 3.97%, 0.69%, and 1.14%, respectively.

3.3 Ablation experiment

We conduct the ablation experiments to evaluate the importance of topology-based and semantic-based contrastive learning modules. Table 3 shows the results of the ablation experiments. In Table 3, 'w/o TCL' means the model removes the topology-based contrastive learning module, 'w/o SCL' means the

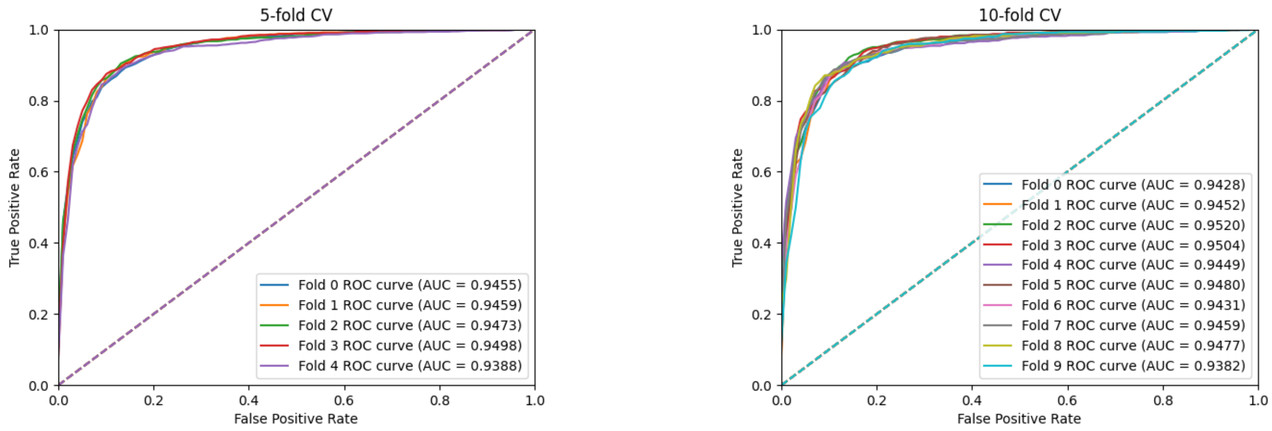

Figure 3. AUC curves of MTCL-MDA on 5(10)-fold cross-validation.

Table 3. Results of ablation experiments of MTCL-MDA model on HMDD v2.0 (%).

models	AUC	ACC	PRE	REC	F1-score
w/o CL	93.29	85.92	82.82	90.70	86.57
w/o TCL	94.00	86.26	84.73	88.87	86.92
w/o SCL	94.13	86.34	83.79	89.43	86.52
MTCL-MDA	94.56	87.70	85.31	91.09	88.10

model removes the semantic-based contrastive learning module, 'w/o TCL' means the model removes all contrastive learning module. The results show that the model achieved the worst performance when it did not use the contrastive learning module. The performance of the model is improved when it adopts topology-based or semantic-based contrastive learning modules. The best performance occurs when the model adopts both topology-based and semantic-based contrastive learning modules. At this time, the AUC, ACC, PRE, REC and $F1-score$ indicators obtained by the model increased by 1.27%, 1.78%, 2.49%, 0.39% and 1.53% respectively. This proves that topology-based and semantic-based contrastive learning modules can work together and play a positive role in improving model performance.

3.4 Stability evaluation

We designed an experiment to make the model run randomly for 20 rounds, and drew the boxplots of the results of these 20 rounds, as shown in Figure 4. Boxplots can show how scattered a set of data is, detect and display outliers in the data, and clean them up. Based on these results, we could judge and observe the overall distribution of the data. When the data distribution is relatively concentrated, the boxes in the boxplot will be smaller, otherwise the boxes will be larger. When the midline is close to the upper edge of the boxplot, the data is concentrated in the upper half part, and when the midline is close to the bottom edge of the boxplot, the data is concentrated in the lower half part. The boxplot results of each indicator in Figure 4 further demonstrate the stability of our model.

In addition, we constructed parameter experiments to study the sensitivity of model performance to the involved hyperparameters. In the experiment, we kept the remaining parameters consistent and evaluated the impact of hyperparameters α, β_1, β_2, and β_3 on model performance, as shown in Table 4. The results indicate that the performance of the model is not sensitive to the hyperparameters α, β_2, and β_3. Therefore we can set these three hyperparameters relatively easily. In addition, when the hyperparameter β_1 is greater

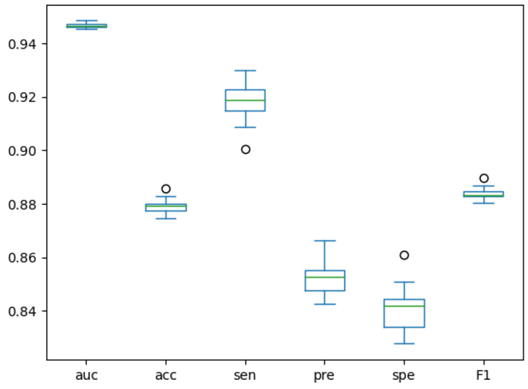

Figure 4. Boxplots of training model for 20 times.

Table 4. Results of parameter experiments of MTCL-MDA model on HMDD v2.0 (%).

Parameters	Rounds	AUC	ACC	PRE	REC	F1-score
	α=0.1	93.56	87.13	84.26	86.56	88.85
β_1=1e-6	α=0.2	93.91	87.19	84.99	89.23	86.84
β_2=1e-8	α=0.5	94.15	88.24	83.58	91.07	88.32
β_3=1e-6	α=1.0	94.31	89.02	84.06	90.98	89.02
	α=2.0	94.18	88.06	83.73	89.23	87.97
	β_1=1e-4	91.13	83.46	81.74	87.48	86.60
α=1.0	β_1=1e-5	91.88	82.41	81.58	83.79	85.16
β_2=1e-8	β_1=1e-6	94.31	89.02	84.06	90.98	89.02
β_3=1e-6	β_1=1e-7	94.29	87.95	85.88	89.04	88.96
	β_1=1e-8	94.04	87.26	85.01	87.29	88.15
	β_2=1e-6	94.24	87.61	84.14	89.96	87.55
α=1.0	β_2=1e-7	94.12	87.42	84.26	89.41	87.33
β_1=1e-6	β_2=1e-8	94.31	89.02	84.06	90.98	89.02
β_3=1e-6	β_2=1e-9	94.19	87.75	84.09	90.33	87.71
	β_2=1e-1	94.26	87.42	84.57	90.24	87.41
	β_3=1e-4	94.27	87.47	84.04	89.78	87.40
α=1.0	β_3=1e-5	94.23	87.75	84.47	89.87	87.66
β_1=1e-6	β_3=1e-6	94.31	89.02	84.06	90.98	89.02
β_2=1e-8	β_3=1e-7	94.35	87.84	84.58	91.16	87.87
	β_3=1e-8	94.28	87.79	84.56	91.07	87.82

than 1e-6, the performance of the model decreases significantly; when β_1 is less than 1e-6, the performance of the model improves significantly and tends to be stable. We also found that when the three parameters β_1, β_2, and β_3 are all set to smaller numbers, the model performance is relatively stable. Therefore, we can set the hyperparameters β_1, β_2, and β_3 to a smaller number, such as 1e-6.

3.5 Case study

To validate the predictive performance of our proposed model MTCL-MDA in practical scenarios, we perform case studies and Table 5 presents the corresponding results. The known biological experiment

Table 5. Top 20 cardiomyopathy-related miRNAs predicted by MTCL-MDA based on HMDD v2.0.

miRNA	miR2Diseas	miRNA	miR2Diseas
hsa-mir-27a	definited	hsa-mir-181b	undefinited
hsa-mir-499a	undefinited	hsa-mir-195	definited
hsa-mir-150	undefinited	hsa-mir-125b	definited
hsa-mir-21	definited	hsa-mir-199b	definited
hsa-mir-1	definited	hsa-mir-27b	definited
hsa-mir-23a	definited	hsa-mir-214	definited
hsa-mir-199a	definited	hsa-mir-23b	definited
hsa-mir-196a	undefinited	hsa-mir-155	definited
hsa-mir-126	undefinited	hsa-mir-9	undefinited
hsa-let-7i	undefinited	hsa-mir-133a	definited

results show that there is some relationship between miRNA and cardiomyopathy. Cardiomyopathy is a relatively serious heart disease. Once patients have symptoms, it will affect their normal life and work. As the disease progresses, the symptoms of heart failure will further aggravate, and symptoms such as edema and dyspnea will appear. The sick will not be able to live and rest normally, and the quality of life will further decline, which will bring heavy burden and pain to the family and society. Patients with hypertrophic cardiomyopathy and arrhythmogenic cardiomyopathy, especially young people, are even at risk of sudden death. Therefore, we selected cardiomyopathy to predict its associated miRNAs.

In case studies, we execute experiments with the following steps: when training the model, firstly, delete the association information between cardiomyopathy (including dilated cardiomyopathy and hypertrophic cardiomyopathy) and miRNA from the 5430 MDAs, and secondly, train the model by randomly collecting corresponding negative samples, and next use the association between cardiomyopathy (including dilated cardiomyopathy and hypertrophic cardiomyopathy) and all miRNAs as test samples. Then output the top 10 miRNAs which are predicted associated with cardiomyopathy. After comparing with the real labels of known samples, it is known that the prediction results are completely correct. This further verifies the superiority of our model. Our model MTCL-MDA has practical implications in the field of mining disease-related miRNAs and providing reliable guidance for disease treatment.

In biological experiments, it has been proved that miRNA is related to various diseases, such as hsa-mir-29a is related to various tumor diseases, including but not limited to breast tumor, liver tumor, gastric tumor and so on. It is likely to be involved in more diseases, so we design experiments to explore whether hsa-mir-29a is interrelated other diseases. The association data between hsa-mir-29a and disease is removed when training the model, and the corresponding negative samples are selected randomly. Finally, we take the association of hsa-mir-29a with all diseases as test samples and output the predicted association probabilities. The predicted results are shown in Table 6, which manifests that miRNAs are not only associated with various tumor diseases, but also involved in Alzheimer's disease (Li et al., 2022a), Parkinson's disease and other mental diseases, some of which have also been verified in the latest database.

4 CONCLUSION

This study propose a model named MTCL-MDA based on graph collaborative filtering, which can accurately predict MDAs. To fully utilize the topological information, we present a contrastive learning strategy based on topological neighborhood. And we designed a contrastive learning strategy based on semantic neighborhood to alleviate the problem of the noise information introduced by the topological

Table 6. Predicted diseases associated with hsa-mir-29a based on MTCL-MDA(TOP 20).

disease	dbDEMC	miR2Diseas
Breast Neoplasms	definited	undefinited
Carcinoma, Hepatocellular	definited	undefinited
Stomach Neoplasms	definited	undefinited
Carcinoma, Hepatocellular	definited	undefinited
Ovarian Neoplasms	definited	undefinited
Mouth Neoplasms	definited	definited
Parkinson Disease	definited	undefinited
Colonic Neoplasms	definited	definited
Crohn Disease	undefinited	undefinited
Stomach Neoplasms	definited	undefinited
Heart Failure	undefinited	undefinited
Schizophrenia	undefinited	undefinited
Colorectal Neoplasms	definited	undefinited
Tuberculosis, Pulmonary	undefinited	undefinited
Aortic Aneurysm, Thoracic	definited	undefinited
Myocardium	undefinited	definited
Melanoma	definited	undefinited
Biliary Atresia	undefinited	undefinited
Liver Cirrhosis	undefinited	undefinited
Endomyocardial Fibrosis	undefinited	undefinited

contrastive learning strategy and the sparse topological neighborhood. The comparison results with the current methods fully demonstrate the high-performance of the MTCL-MDA model. Furthermore, the case analysis suggests that the MTCL-MDA model can be an option for the discovery of disease-associated miRNAs and miRNA-associated diseases, thereby providing constructive suggestions for disease treatment and diagnosis. Since the proposed model uses a lightweight graph collaborative filtering model as the encoder, this may result in the loss of some information during message propagation. In addition, the model will involve relatively complex operations when calculating the contrastive learning loss. In future work, we will focus on solving these problems and improving the applicability of the model.

5 DATA AVAILABILITY

Our data and code are publicly available at: https://github.com/Lqingquan/MTCL-MDA. And our webserver is publicly accessible at: https://huggingface.co/spaces/ZZCrazy00/MDA.

6 COMPETING INTERESTS

There is NO Competing Interest.

7 ACKNOWLEDGMENTS

The work was supported in part by the National Natural Science Foundation of China (No. 62002111, 62172158), the Natural Science Foundation of Hunan Province (No. 2022JJ40090).

REFERENCES

Aitchison, L. (2021). Infonce is a variational autoencoder. *arXiv preprint arXiv:2107.02495*

Ambros, V. (2004). The functions of animal micrornas. *Nature* 431, 350–355

Benes, V. and Castoldi, M. (2010). Expression profiling of microrna using real-time quantitative pcr, how to use it and what is available. *Methods* 50, 244–249

Cai, L., Wang, L., Fu, X., Xia, C., Zeng, X., and Zou, Q. (2021). Itp-pred: an interpretable method for predicting, therapeutic peptides with fused features low-dimension representation. *Briefings in Bioinformatics* 22, bbaa367

Cai, Y., Yu, X., Hu, S., and Yu, J. (2009). A brief review on the mechanisms of mirna regulation. *Genomics, proteomics & bioinformatics* 7, 147–154

Cao, D.-D., Li, L., and Chan, W.-Y. (2016). Micrornas: key regulators in the central nervous system and their implication in neurological diseases. *International journal of molecular sciences* 17, 842

Chen, X., Wang, C.-C., Yin, J., and You, Z.-H. (2018). Novel human mirna-disease association inference based on random forest. *Molecular Therapy-Nucleic Acids* 13, 568–579

Chen, X., Yan, C. C., Zhang, X., You, Z.-H., Deng, L., Liu, Y., et al. (2016). Wbsmda: within and between score for mirna-disease association prediction. *Scientific reports* 6, 21106

Chen, X. and Yan, G.-Y. (2014). Semi-supervised learning for potential human microrna-disease associations inference. *Scientific reports* 4, 5501

Chen, X., Zhu, C.-C., and Yin, J. (2019). Ensemble of decision tree reveals potential mirna-disease associations. *PLoS computational biology* 15, e1007209

Christensen, M. and Schratt, G. M. (2009). microrna involvement in developmental and functional aspects of the nervous system and in neurological diseases. *Neuroscience letters* 466, 55–62

Cuperus, J. T., Fahlgren, N., and Carrington, J. C. (2011). Evolution and functional diversification of mirna genes. *The Plant Cell* 23, 431–442

Ding, Y., Tian, L.-P., Lei, X., Liao, B., and Wu, F.-X. (2021). Variational graph auto-encoders for mirna-disease association prediction. *Methods* 192, 25–34

Friedman, R. C., Farh, K. K.-H., Burge, C. B., and Bartel, D. P. (2009). Most mammalian mrnas are conserved targets of micrornas. *Genome research* 19, 92–105

Ganju, A., Khan, S., Hafeez, B. B., Behrman, S. W., Yallapu, M. M., Chauhan, S. C., et al. (2017). mirna nanotherapeutics for cancer. *Drug discovery today* 22, 424–432

Gao, H., Sun, J., Wang, Y., Lu, Y., Liu, L., Zhao, Q., et al. (2023). Predicting metabolite–disease associations based on auto-encoder and non-negative matrix factorization. *Briefings in Bioinformatics* 24, bbad259

Gebert, L. F. and MacRae, I. J. (2019). Regulation of microrna function in animals. *Nature reviews Molecular cell biology* 20, 21–37

Hill, M. and Tran, N. (2021). mirna interplay: Mechanisms and consequences in cancer. *Disease models & mechanisms* 14, dmm047662

Hu, Y., Deng, H., Xu, S., and Zhang, J. (2015). Micrornas regulate mitochondrial function in cerebral ischemia-reperfusion injury. *International journal of molecular sciences* 16, 24895–24917

Huang, Z., Shi, J., Gao, Y., Cui, C., Zhang, S., Li, J., et al. (2019). Hmdd v3. 0: a database for experimentally supported human microrna–disease associations. *Nucleic acids research* 47, D1013–D1017

James, J. P., Riis, L. B., Malham, M., Høgdall, E., Langholz, E., and Nielsen, B. S. (2020). Microrna biomarkers in ibd—differential diagnosis and prediction of colitis-associated cancer. *International journal of molecular sciences* 21, 7893

Ji, Y., Li, X., Zhu, Y., Li, N., Zhang, N., and Niu, M. (2018). Faecal microrna as a biomarker of the activity and prognosis of inflammatory bowel diseases. *Biochemical and biophysical research communications* 503, 2443–2450

Jiang, Q., Hao, Y., Wang, G., Juan, L., Zhang, T., Teng, M., et al. (2010). Prioritization of disease micrornas through a human phenome-micrornaome network. *BMC systems biology* 4, 1–9

Jiang, Q., Wang, Y., Hao, Y., Juan, L., Teng, M., Zhang, X., et al. (2009). mir2disease: a manually curated database for microrna deregulation in human disease. *Nucleic acids research* 37, D98–D104

[Dataset] Kushary, D. (1998). The em algorithm and extensions

Li, W. and Ruan, K. (2009). Microrna detection by microarray. *Analytical and bioanalytical chemistry* 394, 1117–1124

Li, X., Lin, Y., Meng, X., Qiu, Y., and Hu, B. (2021a). An l_0 regularization method for imaging genetics and whole genome association analysis on alzheimer's disease. *IEEE Journal of Biomedical and Health Informatics* 25, 3677–3684

Li, X., Lin, Y., Xie, C., Li, Z., Chen, M., Wang, P., et al. (2022a). A clustering method unifying cell-type recognition and subtype identification for tumor heterogeneity analysis. *IEEE/ACM Transactions on Computational Biology and Bioinformatics* 20, 822–832

Li, Z., Li, J., Nie, R., You, Z.-H., and Bao, W. (2021b). A graph auto-encoder model for mirna-disease associations prediction. *Briefings in bioinformatics* 22, bbaa240

Li, Z., Zhong, T., Huang, D., You, Z.-H., and Nie, R. (2022b). Hierarchical graph attention network for mirna-disease association prediction. *Molecular Therapy* 30, 1775–1786

Liu, W., Sun, X., Yang, L., Li, K., Yang, Y., and Fu, X. (2022). Nscgrn: a network structure control method for gene regulatory network inference. *Briefings in Bioinformatics* 23, bbac156

Lü, L. and Zhou, T. (2011). Link prediction in complex networks: A survey. *Physica A: statistical mechanics and its applications* 390, 1150–1170

Mirman, D. (2011). Effects of near and distant semantic neighbors on word production. *Cognitive, Affective, & Behavioral Neuroscience* 11, 32–43

Mo, M.-H., Chen, L., Fu, Y., Wang, W., and Fu, S. W. (2012). Cell-free circulating mirna biomarkers in cancer. *Journal of Cancer* 3, 432

Neilson, J. R., Zheng, G. X., Burge, C. B., and Sharp, P. A. (2007). Dynamic regulation of mirna expression in ordered stages of cellular development. *Genes & development* 21, 578–589

Park, J. H., Peyrin-Biroulet, L., Eisenhut, M., and Shin, J. I. (2017). Ibd immunopathogenesis: a comprehensive review of inflammatory molecules. *Autoimmunity reviews* 16, 416–426

Peng, L., Yang, C., Huang, L., Chen, X., Fu, X., and Liu, W. (2022). Rnmflp: predicting circrna–disease associations based on robust nonnegative matrix factorization and label propagation. *Briefings in Bioinformatics* 23, bbac155

Qin, J., Li, R., Raes, J., Arumugam, M., Burgdorf, K. S., Manichanh, C., et al. (2010). A human gut microbial gene catalogue established by metagenomic sequencing. *nature* 464, 59–65

Qu, Y., Zhang, H., Lyu, C., and Liang, C. (2018). Llcmda: a novel method for predicting mirna gene and disease relationship based on locality-constrained linear coding. *Frontiers in genetics* 9, 576

Rendle, S., Freudenthaler, C., Gantner, Z., and Schmidt-Thieme, L. (2012). Bpr: Bayesian personalized ranking from implicit feedback. *arXiv preprint arXiv:1205.2618*

Válóczi, A., Hornyik, C., Varga, N., Burgyan, J., Kauppinen, S., and Havelda, Z. (2004). Sensitive and specific detection of micrornas by northern blot analysis using lna-modified oligonucleotide probes. *Nucleic acids research* 32, e175–e175

Várallyay, E., Burgyán, J., and Havelda, Z. (2008). Microrna detection by northern blotting using locked nucleic acid probes. *Nature protocols* 3, 190–196

Wang, T., Sun, J., and Zhao, Q. (2023). Investigating cardiotoxicity related with herg channel blockers using molecular fingerprints and graph attention mechanism. *Computers in Biology and Medicine* 153,

106464

Wang, W. and Chen, H. (2023). Predicting mirna-disease associations based on lncrna–mirna interactions and graph convolution networks. *Briefings in Bioinformatics* 24, bbac495

Wang, W., Zhang, L., Sun, J., Zhao, Q., and Shuai, J. (2022). Predicting the potential human lncrna–mirna interactions based on graph convolution network with conditional random field. *Briefings in Bioinformatics* 23, bbac463

Weber, J. A., Baxter, D. H., Zhang, S., Huang, D. Y., How Huang, K., Jen Lee, M., et al. (2010). The microrna spectrum in 12 body fluids. *Clinical chemistry* 56, 1733–1741

Weiland, M., Gao, X.-H., Zhou, L., and Mi, Q.-S. (2012). Small rnas have a large impact: circulating micrornas as biomarkers for human diseases. *RNA biology* 9, 850–859

Wu, T., Huang, Q., Liu, Z., Wang, Y., and Lin, D. (2020). Distribution-balanced loss for multi-label classification in long-tailed datasets. In *Computer Vision–ECCV 2020: 16th European Conference, Glasgow, UK, August 23–28, 2020, Proceedings, Part IV 16* (Springer), 162–178

Xu, F., Wang, Y., Ling, Y., Zhou, C., Wang, H., Teschendorff, A. E., et al. (2022). dbdemc 3.0: functional exploration of differentially expressed mirnas in cancers of human and model organisms. *Genomics, Proteomics and Bioinformatics* 20, 446–454

You, Z.-H., Huang, Z.-A., Zhu, Z., Yan, G.-Y., Li, Z.-W., Wen, Z., et al. (2017). Pbmda: A novel and effective path-based computational model for mirna-disease association prediction. *PLoS computational biology* 13, e1005455

Yu, S.-P., Liang, C., Xiao, Q., Li, G.-H., Ding, P.-J., and Luo, J.-W. (2019). Mclpmda: A novel method for mi rna-disease association prediction based on matrix completion and label propagation. *Journal of cellular and molecular medicine* 23, 1427–1438

Zeng, X., Zhang, X., and Zou, Q. (2016). Integrative approaches for predicting microrna function and prioritizing disease-related microrna using biological interaction networks. *Briefings in bioinformatics* 17, 193–203

Zhang, L., Chen, X., and Yin, J. (2019). Prediction of potential mirna–disease associations through a novel unsupervised deep learning framework with variational autoencoder. *Cells* 8, 1040

Zhang, Y., Yun, Z., Gong, L., Qu, H., Duan, X., Jiang, Y., et al. (2018). Comparison of mirna evolution and function in plants and animals. *Microrna* 7, 4–10

Zhou, S., Wang, S., Wu, Q., Azim, R., and Li, W. (2020). Predicting potential mirna-disease associations by combining gradient boosting decision tree with logistic regression. *Computational biology and chemistry* 85, 107200
