# Supplementary figures and images for: An efficient model for predicting human diseases through miRNA based on multiple-types of contrastive learning
Source: Front Microbiol. 2023 Dec 14;14:1325001. doi: 10.3389/fmicb.2023.1325001 (PMC10755968; doi:10.3389/fmicb.2023.1325001)

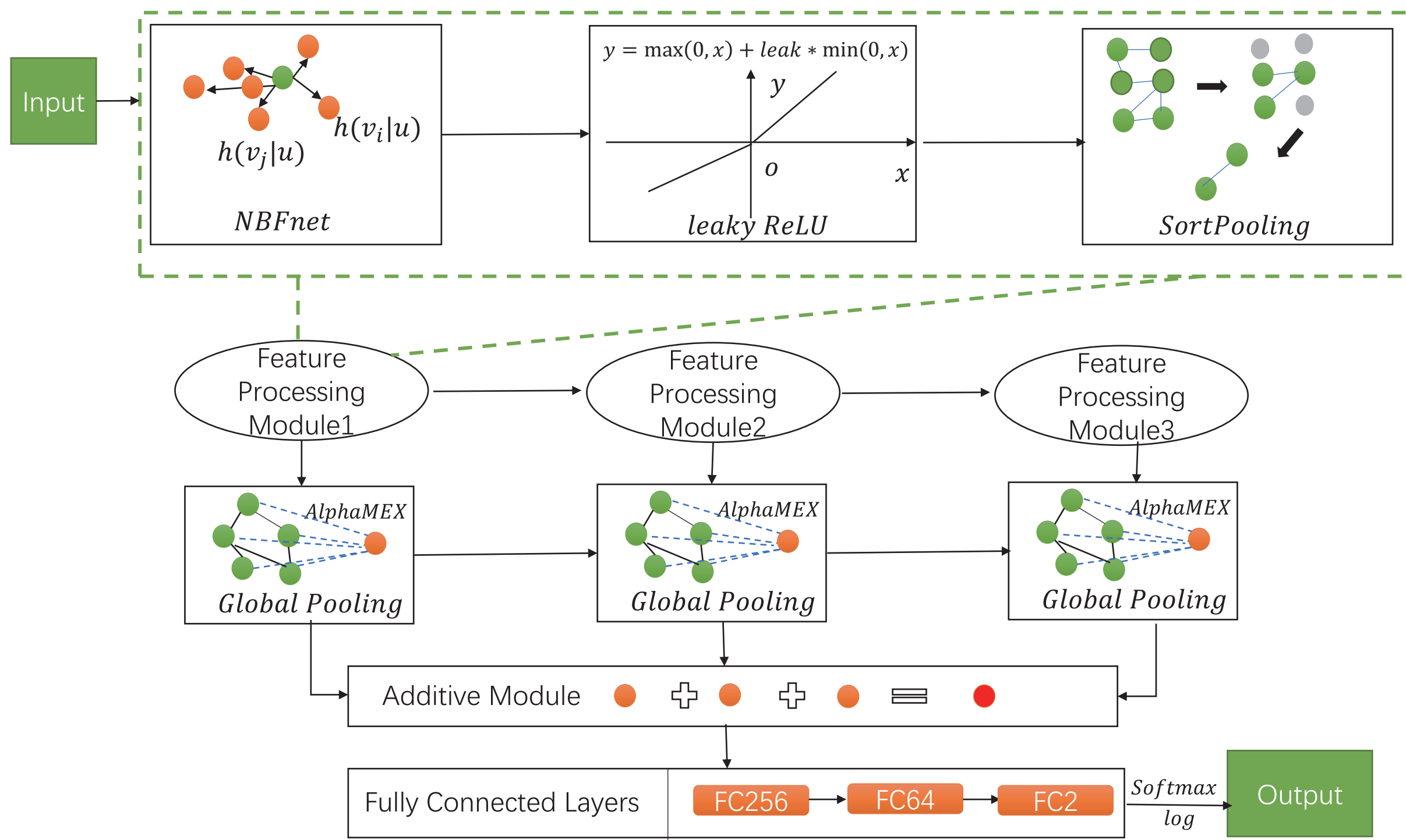

Supplement: Supplementary file 1 [file Data_Sheet_1.ZIP › MTCL-MDA/Figure1-eps-converted-to.pdf]

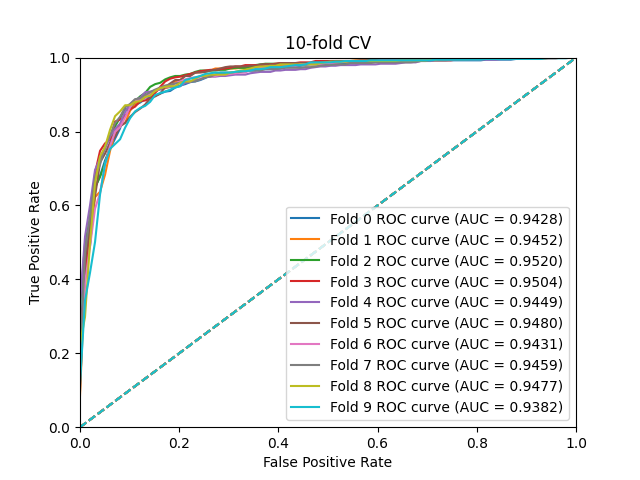

Supplement: Supplementary file 1 [file Data_Sheet_1.ZIP › MTCL-MDA/file/10-fold.png]

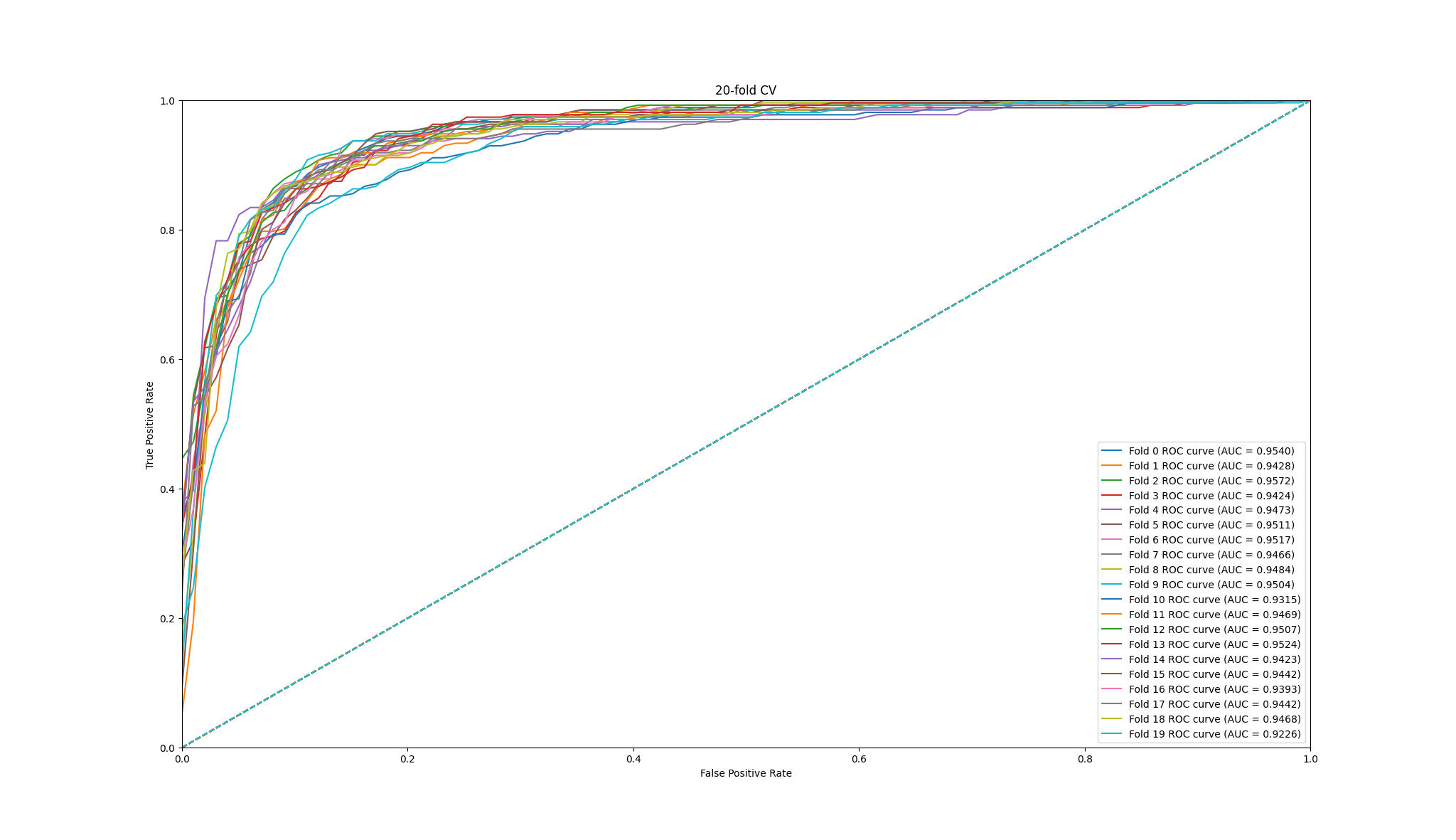

Supplement: Supplementary file 1 [file Data_Sheet_1.ZIP › MTCL-MDA/file/20-fold.png]

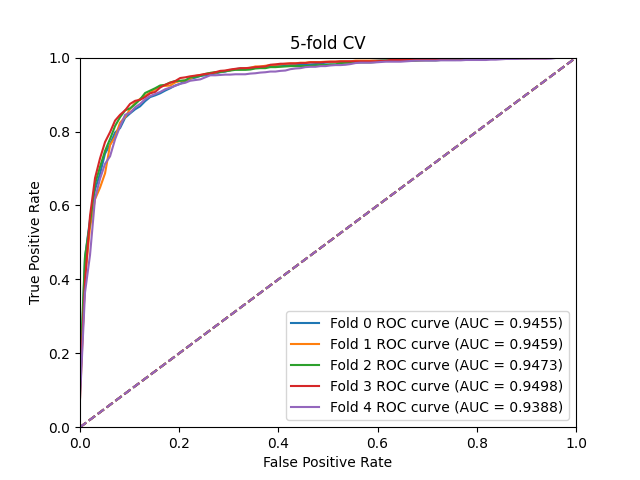

Supplement: Supplementary file 1 [file Data_Sheet_1.ZIP › MTCL-MDA/file/5-fold.png]

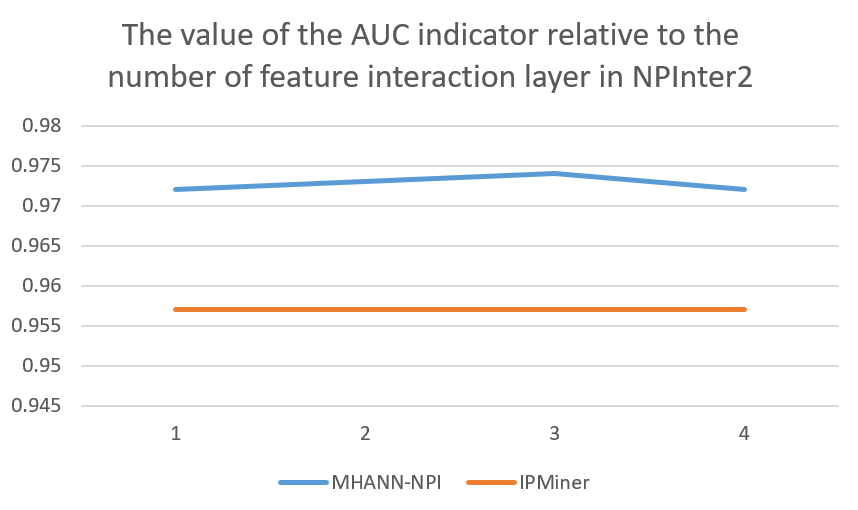

Supplement: Supplementary file 1 [file Data_Sheet_1.ZIP › MTCL-MDA/file/att_layer_num_NPInter2.png]

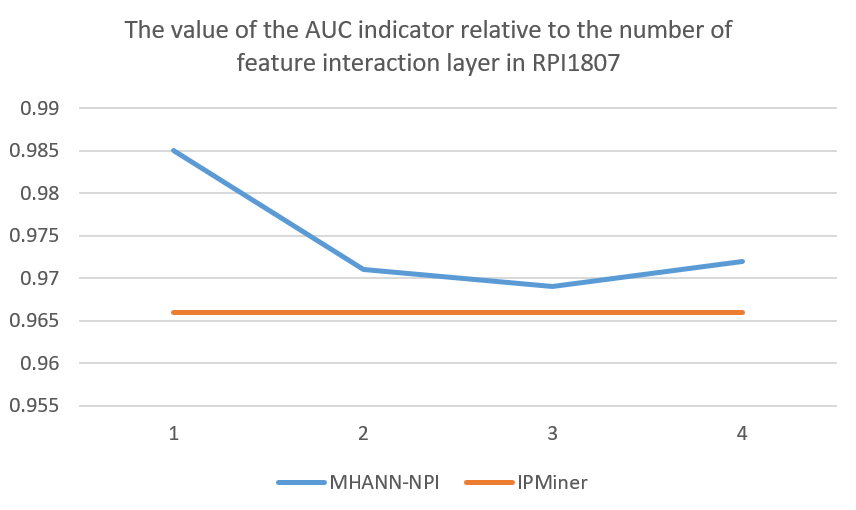

Supplement: Supplementary file 1 [file Data_Sheet_1.ZIP › MTCL-MDA/file/att_layer_num_RPI1807.png]

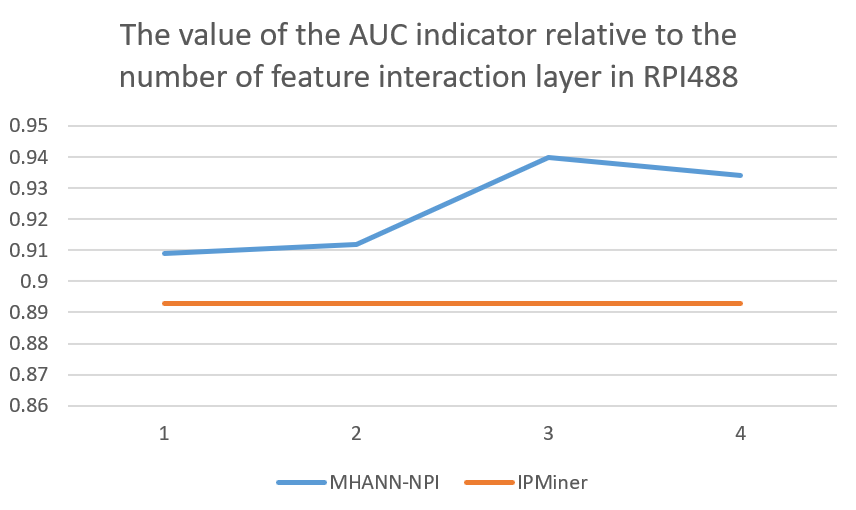

Supplement: Supplementary file 1 [file Data_Sheet_1.ZIP › MTCL-MDA/file/att_layer_num_RPI488.png]

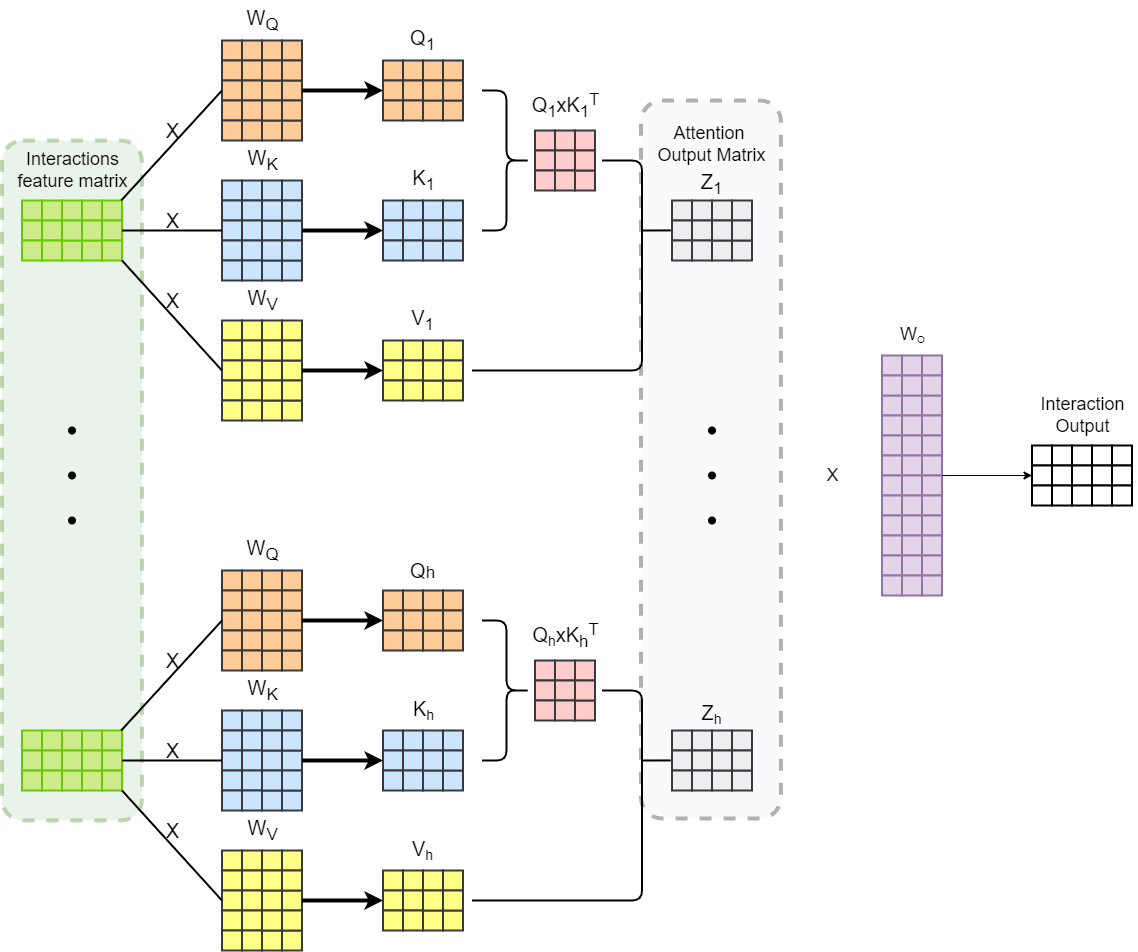

Supplement: Supplementary file 1 [file Data_Sheet_1.ZIP › MTCL-MDA/file/attention.png]

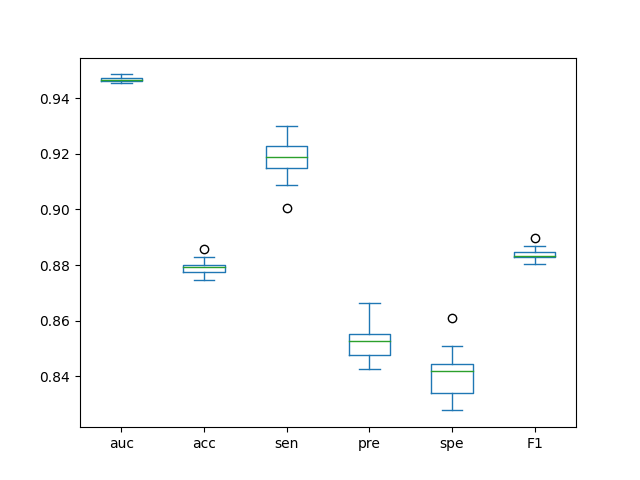

Supplement: Supplementary file 1 [file Data_Sheet_1.ZIP › MTCL-MDA/file/box.png]

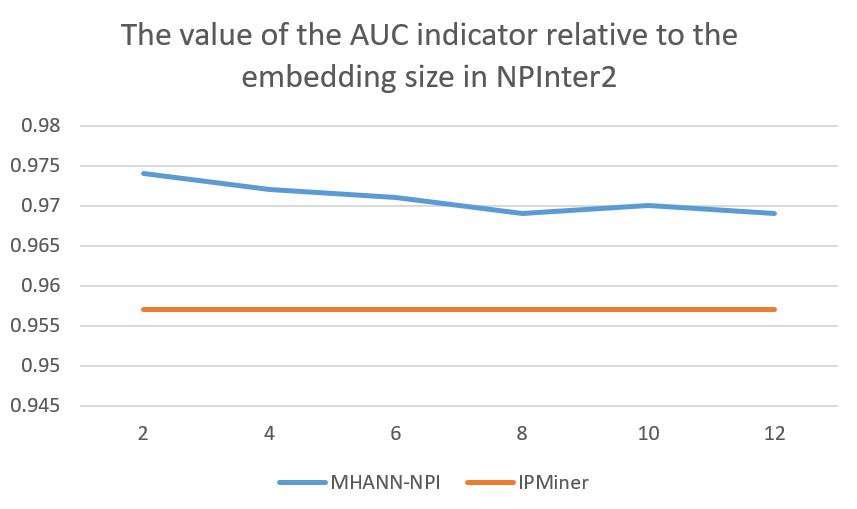

Supplement: Supplementary file 1 [file Data_Sheet_1.ZIP › MTCL-MDA/file/embedding_size_NPInter2.png]

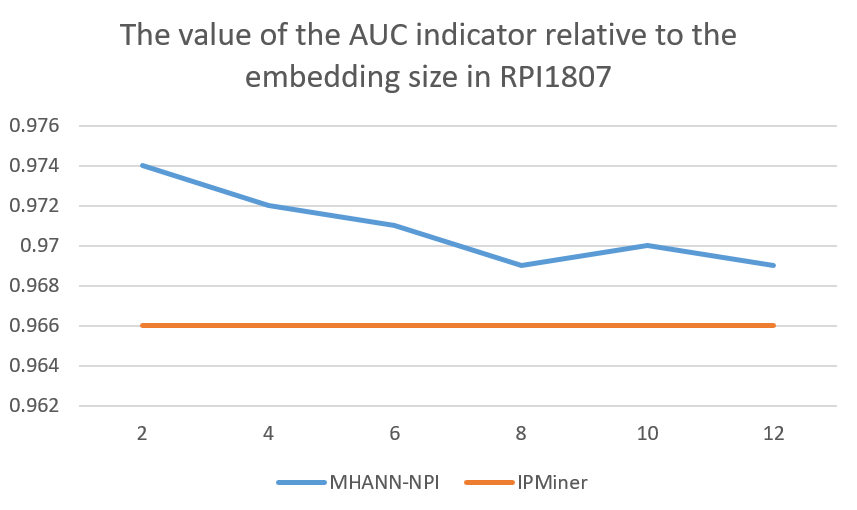

Supplement: Supplementary file 1 [file Data_Sheet_1.ZIP › MTCL-MDA/file/embedding_size_RPI1807.png]

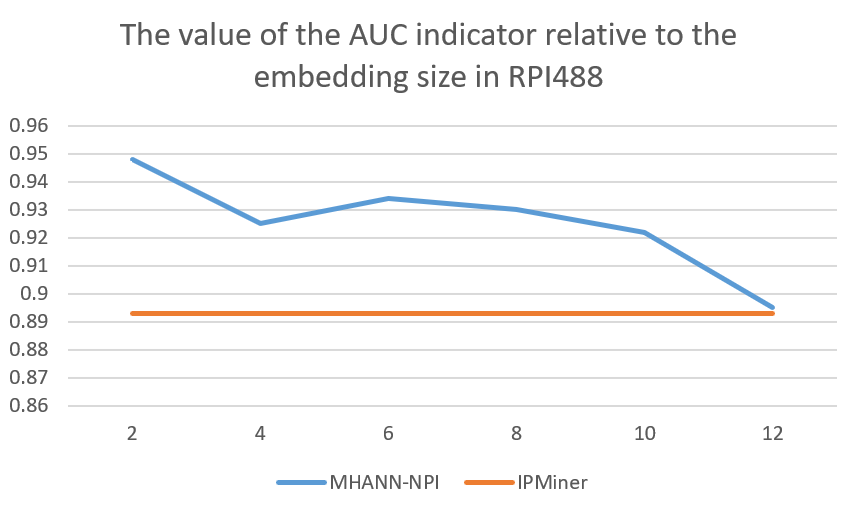

Supplement: Supplementary file 1 [file Data_Sheet_1.ZIP › MTCL-MDA/file/embedding_size_RPI488.png]

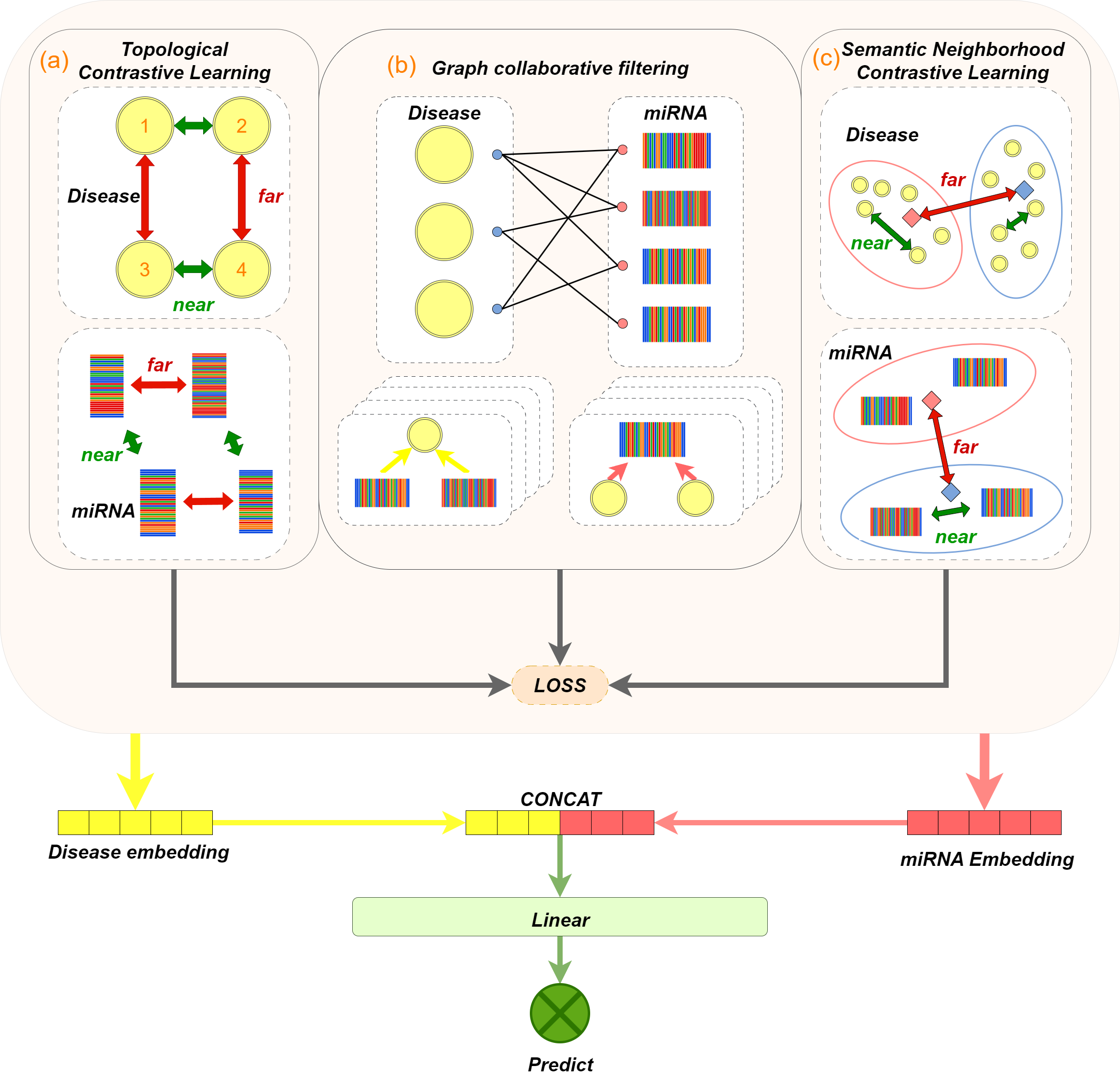

Supplement: Supplementary file 1 [file Data_Sheet_1.ZIP › MTCL-MDA/file/model.png]

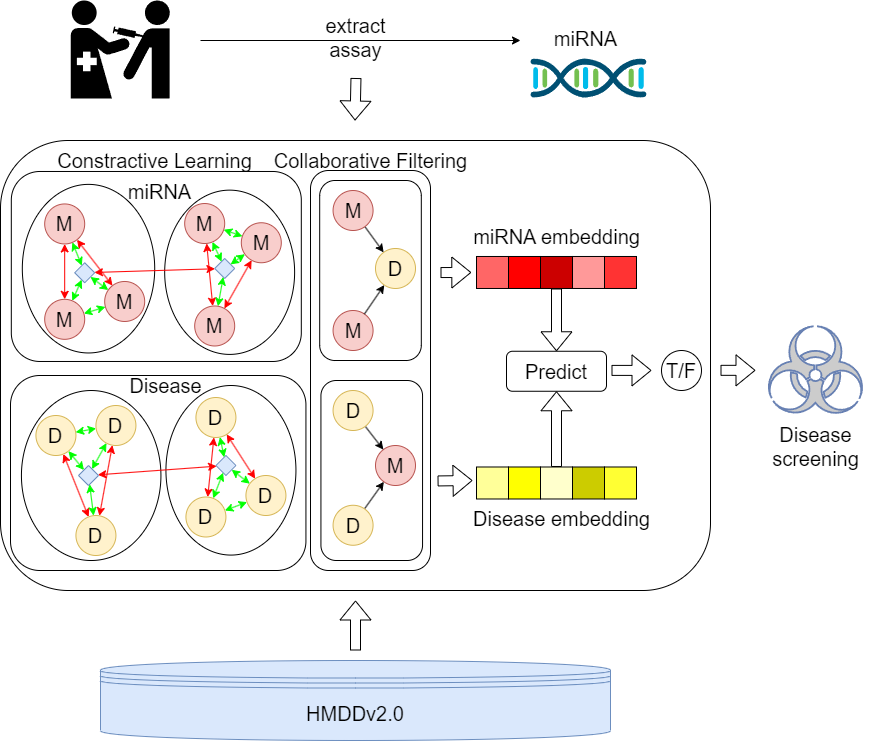

Supplement: Supplementary file 1 [file Data_Sheet_1.ZIP › MTCL-MDA/file/screen.png]

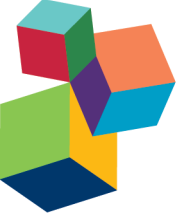

frontiers

Supplement: Supplementary file 1 [file Data_Sheet_1.ZIP › MTCL-MDA/logo1-eps-converted-to.pdf]
